# Controlled Synthesis of Luminescent Xanthene Dyes and Use of Ionic Liquid in Thermochromic Reaction

**DOI:** 10.3390/molecules27103092

**Published:** 2022-05-11

**Authors:** Bartłomiej Potaniec, Maria Zdończyk, Joanna Cybińska

**Affiliations:** 1 Advanced Materials Synthesis Group, Materials Science & Engineering Center, Łukasiewicz Research Network—PORT Polish Center for Technology Development, Stabłowicka 147 Street, 54-066 Wrocław, Poland; bartlomiej.potaniec@port.lukasiewicz.gov.pl (B.P.); maria.zdonczyk@chem.uni.wroc.pl (M.Z.); 2 Faculty of Chemistry, University of Wroclaw, F. Joliot-Curie 14 Street, 50-383 Wrocław, Poland

**Keywords:** fluoran leuco dye, xanthene dyes, luminescence, synthesis optimization, thermochromic materials, ionic liquid

## Abstract

In this study, we demonstrate six novel xanthene derivatives and their spectroscopic and chemical properties. The presented synthesis examination allowed us to obtain two different compounds during one step, with open and closed lactone rings substituted with different length alkyl chains. Increasing the reaction efficiency to 77% was obtained using the microwave-assisted method. Moreover, the modification of *O*-alkylation synthesis in an ecofriendly way using a ball mill led to achieving exclusively one opened ring product. All of the synthesized compounds showed different spectroscopic behaviors in comparison with the different organic dyes; the typical concentration quenching of luminescence was not observed. The relationship between the length of the alkyl chain and the time of luminescence decay is presented. Synthetized closed forms of dyes turned out to be promising leuco dyes. For the first time, an ionic liquid was used as a developer of synthesized xanthene derivatives (as leuco dyes), which led to obtaining an irreversible thermochromic marker.

## 1. Introduction

Today’s technology and photonics are based on newer and newer organic dyes with high luminescent properties. Together with good optical properties, these dyes must also be chemically and thermally stable, especially as they often are incorporated into sol-gel matrices during the preparation of hybrid platforms. One possible answer to modern requirements could be xanthene dyes and their derivatives [1]. They have found applications in bioimaging [2,3,4,5,6,7], photonic [8,9,10,11] as well as optic sensors [12,13,14,15,16]. Although they have excellent spectroscopic properties (e.g., high quantum yields ~95%), some problems are also noticeable. They may photodecompose during heating [17], having relatively short luminescence lifetimes (around 4 ns) [18] for imaging applications [19,20,21,22,23,24]. It is, however, possible to modify the dye structure to change the luminescent properties (absorption and emission ranges). Generally, xanthene dyes contain two different Π-systems, which are phenyl and xanthene. Crystal structure Π-systems arrange themselves perpendicular to each other due to the weak interactions between the electron clouds. However, when in a solution, the situation is different due to phenyl system rotation [18]. As a result, a modification in one of the Π-electron systems may cause changes in the luminescent properties. Additionally, a strong dependence of the dye behavior in various solvents is observed [25,26].

To achieve our aim of creating new functional materials, we took up the challenge of synthesizing high-luminescent derivatives of fluoran. It should be stated that the xanthene dye group can be divided into structural classes, e.g., fluorans and spirofluorenes. They can be synthesized in two forms—closed (3′,6′-substitution) and open—ether-ester-like (2,6′-substitution). Both forms can be obtained in a relatively simple way as a result of a maximum of two-step synthesis [27,28,29]. In recent years, synthetic routes have been developed to obtain both forms of the xanthene dye through one reaction, with yields up to 84% as a result of a single-step synthesis [30]. Since organic dyes can be synthesized using the classical method, such as heating in a heating mantle, nowadays, modern methods of synthesis are increasingly used. The use of a microwave reactor allows for homogeneous heating of the reaction mixture. This procedure allows the reaction time to be shortened by up to 10 min while maintaining high (91%) reaction yields [31,32,33]. An additional advantage is a reduction in the number of by-products, especially in solvent-free reaction environments [34,35,36,37]. In this work, we present both classical and microwave-heating-assisted methods in order to compare reaction efficiency. Furthermore, in our study, an interesting alternative is the development of ball-milling synthesis in the presence of a proper catalyst, which aligns perfectly with the trend toward green chemistry [38,39,40]. 

**Figure 1:**
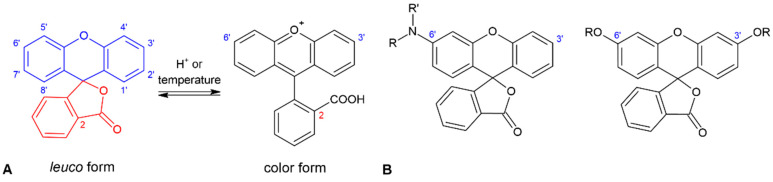
Leuco and color form of fluoran (**A**) and examples of typical substitution of fluoran-based leuco-dyes (**B**).

As is well known, xanthene dyes are also able to act similarly to leuco dyes. Leuco dyes are a group of compounds in which the transition from a colorless form (*leuco* form) to a colored one is a result of a chemical or photochemical reaction of the opening of a spironolactone ring [41,42,43]. The reaction can be reversible or irreversible (Figure 1A). Fluorans can therefore be considered functional compounds that can be used in thermo-, pH−, or pressure-sensitive recording systems [10,20,44,45,46]. The main two groups of leuco dyes based on fluoran skeletons possess different groups attached in position 6′ or positions 3′ and 6′ (Figure 1B). Both the starting color of the leuco dye and the developed color depend on the type and number of substituents attached to the parent structures. 

During the research, we paid attention to the synthesized closed products, whose spectroscopic, as well as thermochromic properties (mainly color change in the developer during heating) showed a wide application potential, e.g., in overheating sensors. This family group shows different spectroscopic properties in comparison to classical organic dyes. It should be stressed that the luminescence efficiency of the newly synthetized derivatives did not decrease with the increasing concentration, which is different than what is expected for organic systems. Synthesized di-*O*-alkylated closed forms of fluorans changed color to yellow, orange, or even red after mixing with the ionic liquid (IL) and subsequent heating. Moreover, it was noticed that after modifying the length of the attached alkyl chain, the transformation temperature, final colors, and intensity varied. However, we did not test for the selection of the appropriate developer (the acid), because we chose ionic liquid (IL) as the new developer. To the best of our knowledge, it is the first time using IL in such systems.

## 2. Results and Discussion

### 2.1. Controlling Synthesis

**Figure 2:**
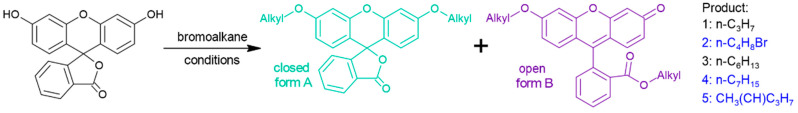
Preparation scheme of long chain alkyl derivatives (product **A** (3′,6′-dialkyloxy derivative) —closed lactone ring; product **B** (2,6′-ether–ester)—opened lactone ring) (top) with magnification on new, long alkyl chain fluorans (bottom). Conditions: Cs_2_CO_3_, DMF, 70 °C, 24 h for classical heating method; Cs_2_CO_3,_ DMF, 70 °C, 10 min for microwave-assisted method; Cs_2_CO_3,_ TBAB, 18-crown-6, 1 h for ball-mill assisted method.

The literature reports on the preparation of the 3′,6′-di-*O*-alkyloxy closed derivative and the 2,6′-ether-ester open derivative as a result of the reaction of the 3′,6′-dihydroxyfluoran with sodium hydroxide or potassium carbonate in methanol or *N*,*N*-dimethylformamide (DMF) environment [28,47,48]. However, in each case, only one derivative was obtained, from the second derivative was synthesized in the next step [28,49]. In comparison, we synthesized two different luminescent and thermochromic new fluoran derivatives in one synthesis step. This allowed us to significantly minimize and simplify the steps needed to obtain the final products. This was possible with apolar DMF (in which the cesium carbonate base dissolves well). We chose this solvent for the reaction according to our previous studies concerning *O*-alkylation’s reactions [50,51,52,53]. Moreover, the syntheses using dimethyl sulfoxide (DMSO) or 1-methyl-2-pyrrolidinone (NMP) as a solvent resulted in lower reaction yields. Moreover, the time of the reaction, 24 h, was considered optimal for maximizing the resultant yields because we did not observe a significant increase in reaction yield when extending the time to 96 h. In this work, to the best of our knowledge, we present derivatives **2**, **4**, and **5** (both closed **A** and open **B**), which have not been synthesized before in earlier research (Figure 2). *N*-propyl, 1-bromobutyl, n-hexyl, n-heptyl, or 1-methylbutyl chains were used for the reaction.

The synthesis that used classical heating, despite good efficiency, was too long, as it lasted 24 h. In order to shorten the reaction time as well as increase the reaction yield, we chose to adopt the previously used conditions using a microwave reactor. This reaction is interesting in that it allows one to exclude the use of solvents, which aligns with the green chemistry trend. 

**Figure 3:**
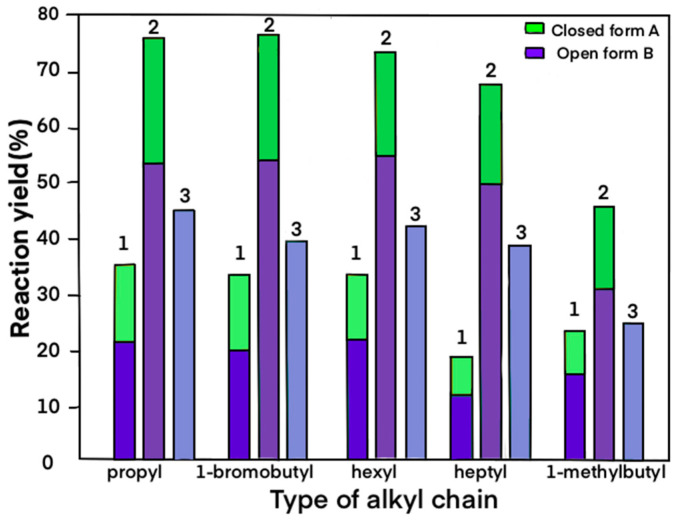
Reaction yields of synthesized derivatives using different methods. The graph shows columns 1 as classic heating, 2—microwave assisted method and 3 as a ball mill (dark and light green colors—products **A**, dark and light purple colors—products **B**).

When using the classical *O*-alkylation method, the observed overall yields of the reaction were within the maximum range of 19–35% in the case of using DMF as the solvent and Cs_2_CO_3_. Alkylation reactions carried out in DMSO or NMR resulted in much lower overall yields, up to a maximum of 17%. It is worth noting that the carbon chain elongation did not affect the reaction yields in the case of derivatives **1**–**3** (~35%). The lower yields were only observed for products **4** (19%) and **5** (24%), probably due to the sterical hindrance of the attached alkyl chain. Due to the sustainable use of substrates, as well as the effectiveness of separation and purification of the resulting products, we tried to find the ratios of both **A** and **B** forms of each product as close to 1:1 as possible. The microwave-assisted method allowed us to reduce the required time to 10 min with a simultaneous increase in efficiency up to 77% (sum of both products). The reaction time of 10 min in the microwave reactor was the shortest time tested which allowed us to obtain the best yields. Extending the reaction to 30 min did not significantly increase the amount of the obtained products. The greatest increase in overall reaction yield (from 19% to 68%) was observed in the case of the substitution with n-heptyl chain derivative **4**. Figure 3 shows a comparison of the methods used, distinguishing between the open and closed forms. In the case of both classical heating and microwave heating, the open form of **B** is the dominant product. Despite the increase in the reaction yield, the ratio between each of the forms remained unchanged.

The reactions in the ball mill were carried out to obtain luminescent open product **B** only. The use of a ball mill or even a mortar can lead to opening the ring due to the mechanical force involved [54]. In the case of **1B**, the reaction was carried out with Cs_2_CO_3_ without a catalyst and had an overall yield of 10%. The addition of 18-crown-6 or tetrabutylammonium bromide (TBAB) raised this efficiency to only 28%. The highest amount of product **1B** was obtained with the simultaneous application of 18-crown-6 and TBAB—45%. Using a base—Cs_2_CO_3_ and both phase transfer catalysts—TBAB and 18-crown-6 (0.1 eq of each simultaneously) makes it possible to achieve the desired product in 1 h. Extending the reaction time did not result in significant changes in its yield. In this method, the products were obtained with higher yields in comparison to classical heating while shortening the duration of the reaction in an environmentally friendly way. Using a ball mill allows us to have a comparable open yield product to microwave-assisted synthesis. The greatest increase in synthesis yield, by 3.25-times, was observed for the reaction of the new fluoran derivative **4**.

### 2.2. Spectroscopic Properties

**Figure 4:**
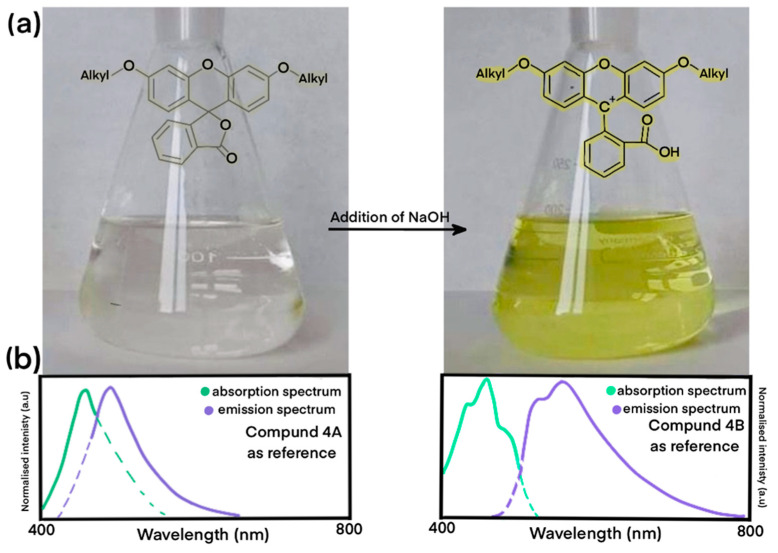
(**a**) Changes in color of solution of compounds **A** after adding NaOH with probable chemical formula for both forms (before and after addition of base); (**b**) Emission and absorption spectra of compounds **4A**, **4B** as references.

The derivatives in both forms differ significantly in their spectroscopic properties, and to compare them, detailed measurements for absorption, emission, quantum yields, and decay times were made. All of the data were collected and are presented in Table S1 in the Supplementary Materials Section S2. To distinguish between the forms, we will continue the nomenclature as form **A** (closed ring fluoran, low-luminescent) and form **B** (open ring fluoran, highly luminescent). All of the luminescent measurements for the open-ring fluorans were carried out in 0.1 M of NaOH as well as in acetonitrile (ACN), although a longer alkyl chain caused the solubility to deteriorate (Figure S41, Supplementary Materials). For the closed-ring fluorans, measurements were carried out in can only. Analyzing the spectroscopic properties of the closed-form compound **A**—absorption spectra are definitely lower quality with slightly outlined peaks. For both forms, when comparing the emission and absorption spectra, it can be clearly seen that they are not mirror images of each other. The reference spectra are shown in Figure 4b. It is worth noting that in the absorption spectra, we noticed slight shifts towards higher energies; however, the most important factor for the position of the absorption bands is whether the form is open or closed. In the case of synthesized derivatives, chain elongation does not significantly affect the position of the absorption maxima. For open form **B**, absorption spectra consist of two maxima of appropriately equal intensity at 455 nm and 475 nm. Moreover, the shoulder at around 420 nm is visibly associated with the xanthene ring [55,56]. For closed forms **A**, wide bands with one maximum can be observed. Taking into account the fact that derivative **A** does not have luminescent properties, even in the solvent ACN, we must observe the change to form because the emission at excitations around 440 nm is visible. 

In the case of organic solvents, in our example (ACN), the color change of the solution is not as visible as with the addition of an acid/base. In Figure 4a we have shown the probable structures found in ethanol and ethanolic solution of NaOH. The addition of NaOH to the solution with closed lactone ring products should lead to the opening of the lactone ring [57]. 

**Figure 5:**
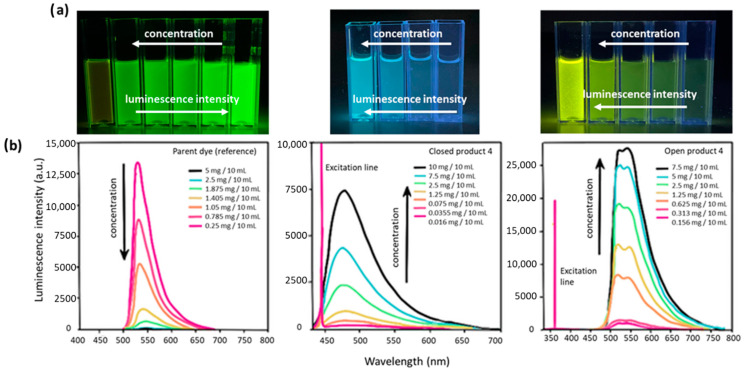
(**a**) Photographs of obtained solutions with different concentrations under excitation with UV lamp (λ_ex_ = 360 nm). From left: fluorescein, compound **4A**, compound **4B**; (**b**) Emission spectra of synthesized closed and open compounds **4A** and **4B** in comparison to fluorescein. Spectra for open product and reference fluorescein were registered in 0.1 M NaOH, while closed form in ACN. For reference and open product were recorded with λ_ex_ = 360 nm and λ_ex_ = 455 nm for closed one.

The electronic spectra of the synthesized fluorans show different spectroscopic behavior than the parent fluorescein (Figure 5). Measurements of the highly concentrated solutions for both fluorescein and synthesized dyes were carried out. The recorded emission spectra for the fluorescein solutions with a concentration higher than 0.25 mg/10 mL indicate that the intensity of luminescence decreases with the increasing concentration. The opposite situation is observed in the solutions of the novel synthesized luminescent dyes. Moreover, it should be mentioned that all of the newly synthesized compounds show similar behaviors (Figure 5).

**Figure 6:**
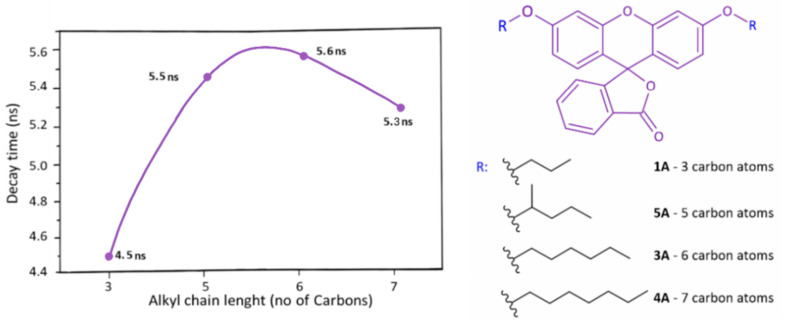
Decay times as function of alkyl chain length for derivatives **A**.

The measurements of the quantum yields using an integrated sphere show results between about 10 and 30% for derivatives **A**, while for **B**, the results are 25 to 35% (Table S1, Supplementary Materials). The lifetimes of the luminescence measurements were prepared for all of the synthesized compounds, and the detailed values are included in Table S1, Supplementary Materials. One can notice that in the case of **A** derivatives, increasing the length of the alkyl chain extends the decay times up to the hexyl chain, and then it shortens (Figure 6) taking a shape similar to a Gaussian curve. For derivatives **B**, all decay times were calculated around ~3.5 ns, and no relationship was found between its value and the length of the alkyl chain. When comparing the values that we obtained in the experiment with the literature values for fluorescein derivatives, the possible opening of the lactone ring, as well as the different distribution of charges in different forms, should be taken into account. A parent dye can occur in five forms that are strongly dependent on the environment. When comparing the data obtained in the experiment with the literature values for fluorescein derivatives, the possible opening of the lactone ring, as well as the different distribution of charges in different forms, should be taken into account. It can be seen that the obtained results resonate with the data gathered from the literature on the cationic form (in the case of A derivatives measured in solution) and the neutral form (in the case of **B** derivatives) [58].

### 2.3. Thermochromic Properties

The tested thermochromic materials were formed from the junction between the two components: (1) a synthesized compound, which acts as an electron acceptor (2) and an ionic liquid that is capable of donating electrons. So far, it has been proven that the combination of fluorescein and IL can be used as a fluorescent polymerizable imidazolium-based ionic liquid for photonic application [59], but to the best of our knowledge, IL for any kind of leuco dyes systems that we have so far encountered do not make use of an ionic liquid as a developer.

The typically observed effect in the literature shows the *leuco* system at the lower temperature as a dark red/orange, which discolors when heated [60]. In the case of the classically selected components of the mixture, the reaction is reversible [61]. However, focusing on the potential applications as sensors for overheating, defrosting, etc., it is essential that the color change cannot be reversed. The mixture of the ionic liquid powder and the synthesized closed-form after melting is colorless, while during the continued heating, it irreversibly turns an orange-red color. 

As a starting point, we used both fluoran derivative **A** and the ionic liquid in a powdered form. We mixed them in a ratio of 1:1 directly before use on a glass substrate. Then we heated the system in a closed heating block with a constant heating rate of 20 °C/min. 

**Figure 7:**
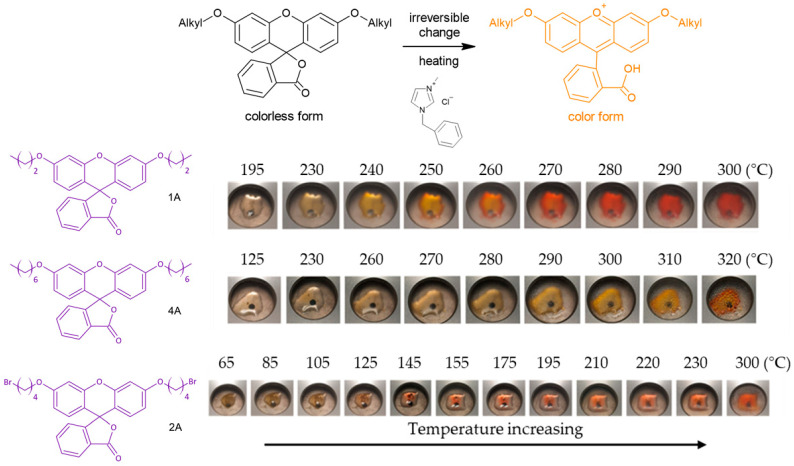
Scheme of thermochromic reaction of fluoran derivatives and IL (top) and changes of color during heating of fluoran derivative-IL mixtures.

During the examination, we observed a correlation between alkyl chain length, the temperature at the beginning of the color change, and the final color development (Figure 7). The greatest change is visible in the first system, which contains propyl substituted fluoran (**1A**) as an acceptor and IL (1-benzyl-3-methylimidazolium chloride, [BMIm][Cl]) as an electron donor. The system with the weakest color change had a bromo-substituted alkyl chain in its system (**2A**), which delayed the color change. For the derivatives substituted with the longest heptyl chains (**4A**), a color change to orange was observed. During the heating process, we observed the development of an intense yellow/orange-red color as a consequence of the ring-opening of the lactone ring through the intermolecular association between the ionic liquid and the spironolactone units. In the case of compounds **1A** and **4A,** possessing electron-donating groups were observed to change color upon the irreversible development from colorless to orange. On the other hand, compound **2A**, with bromine atoms of an electron-accepting nature, changed its color irreversibly to orange-red. Similar observations in the case of the reversible color changes induced by fluorans depending on the nature of the attached substituent were observed in the work of Oh et al. [54]. In addition, the use of a different IL in future studies, as well as a change in the ratio of the compound to IL, will allow us to obtain systems with different transition temperatures and color intensities.

## 3. Materials and Methods

### 3.1. General Methods

All of the organic solvents and reagents for synthesis were purchased from proper commercial sources and used directly.

Crude products of the syntheses were purified by liquid column chromatography using silica gel 60 (40–63 mesh) and a mixture of chloroform:methanol 10:1 (*v*/*v*) as an eluent. The progress of the reactions was monitored by TLC (thin layer chromatography) using Macherey-Nagel silica gel Polygram SIL G plates (Macherey-Nagel GmbH & Co., Düren, Germany). The compounds on the TLC plates were visualized with UV light (λ = 254 nm). The final purity of the compounds (>95%) was checked by HPLC (high-performance liquid chromatography) using the Varian ProStar 210 (Agilent, Santa Clara, CA, USA) with a dual λ absorbance detector system equipped with: Method A—Discovery^®^ BIO Wide Pore C8 HPLC Column (10 μm particle size, 25 cm × 21.2 mm) (Sigma Aldrich, Darmstadt, Germany) and the flow rate was set as 15 mL/min with a gradient of 5−95% (0.05% of TFA (trifluoroacetic acid) in acetonitrile) in (0.05% TFA in water) over 15 min or Method B—Discovery^®^ BIO Wide Pore C8 HPLC Column (10 μm particle size, 25 cm × 4.6 mm) with flow rate at 0.9 mL/min with gradient 0−100% (0.05% of TFA in acetonitrile) in (0.05% of TFA in water) over 15 min.

All the structures of the obtained compounds were determined using nuclear magnetic resonance (NMR). The spectra were measured on a Bruker Avance 600 MHz (600.58 MHz for ^1^H NMR, 150.92 for ^13^C NMR) spectrometer (Bruker, Billerica, MA, USA). Chemical shifts were expressed as parts per million (ppm) relative to internal standard—tetramethylsilane (TMS). 

Infrared spectra (FT-IR) were determined using a Tensor 27 FTIR spectrometer (Bruker, Billerica, MA, USA) equipped with an ATR accessory with a diamond crystal in the wavelength range of 400–4000 cm^−1^.

Positive-ion HR ESI-MS spectra were measured on a Bruker ESI-Q-TOF Maxis Impact Mass Spectrometer (Bruker, Billerica, MA, USA). For the direct infusion of ESI-MS parameters, the mass spectrometer was operated in a positive ion mode with the potential between the spray needle and the orifice as 3.5 kV, a nebulizer pressure of 0.4 bar, and a drying gas flow rate of 3.0 L/min at 200 °C. The sample flow rate was 3.0 µL/min. Ionization mass spectra were collected at the ranges of *m*/*z* 50–1250. The samples were dissolved in methanol and diluted 100-fold and 1000-fold with acetonitrile containing 0.1% of formic acid. For calibration, a calibration solution with sodium formate clusters in the mass range of 50–1250 *m*/*z* was used.

The absorption spectra were measured at room temperature on a Varian Cary 5000 Scan spectrophotometer (Agilent, Santa Clara, CA, USA) in the range of 500–400 nm. The measurements of emission and excitation spectra, in addition to the luminescence quantum yields, were recorded at room temperature on an Edinburgh Instruments FLS980 spectrofluorometer (Edinburgh Instruments, Livingston, UK) equipped with a xenon lamp. During the measurements of the photonic layers, optical filters were used. The decay time measurements were recorded on an Edinburgh Instruments FLS980 spectrofluorometer equipped with 280 and 360 nm laser diodes.

The melting point temperatures were investigated under a polarized optical microscope, DM2700P (Leica Microsystems GmbH, Wetzlar, Germany), equipped with a heating/cooling stage LTS420 (Linkam Scientific Instruments Ltd., Salfords, UK). The samples that were enclosed between two microscopic slides were placed on the Peltier stage of the heating/cooling stage, and the changes were observed under a microscope. The melting temperatures were determined in the heating cycle at a heating rate of 10 °C/min.

Thermochromic properties were measured using the heating stage LTS420 (Linkam Scientific Instruments Ltd., Salfords, UK). The thermochromic test systems were prepared by carefully mixing the appropriate ingredients in a vial. The homogeneous mixtures prepared in this way were put on a glass slide and examined on a heating stage under a microscope. The samples were heated at a rate of 20 °C/min. 

### 3.2. General Procedure of Synthesis

#### 3.2.1. Classical Synthesis (Method A)

Fluorescein (1 g, 3 mmol, 1.0 eq) and Cs_2_CO_3_ (3.9 g, 12 mmol, 4 eq) were dissolved in 5 mL of DMF and vigorously stirred. Then, the proper alkyl bromide was added, and the stirring was continued for the next 24 h under a nitrogen atmosphere at 70 °C. After the completion of the reaction, 30 mL of ethyl acetate was added to the final mixture, and the combined phases were washed with 25 mL of 5% citric acid. The combined aqueous phases were extracted with 3 portions of 25 mL of ethyl acetate. Finally, the organic phases were collected, washed with brine, and dried over Na_2_SO_4_. The mixture was filtered, and the solvent was evaporated to yield the crude products as an orange solid, which was then purified via column chromatography using silica gel as a stationary phase. 

#### 3.2.2. Microwave Synthesis (Method B)

Fluorescein (1 g, 3 mmol, 1.0 eq) and Cs_2_CO_3_ (3.9 g, 12 mmol, 4 eq) were dissolved in 5 mL of DMF and vigorously stirred. Then, the proper alkyl bromide was added, and the stirring was continued for the next 10 min in a microwave reactor under a nitrogen atmosphere at 70 °C. After the completion of the reaction, 30 mL of ethyl acetate was added to the final mixture, and the combined phases were washed with 25 mL of 5% citric acid. The combined aqueous phases were extracted with 3 portions of 25 mL of ethyl acetate. Finally, organic phases were collected, washed with brine, and dried over Na_2_SO_4_. The mixture was filtered, and the solvent was evaporated to yield the crude products as an orange solid, which was then purified via column chromatography using silica gel as a stationary phase. 

#### 3.2.3. Ball-Milling Synthesis (Method C)

Fluorescein (1 g, 3 mmol, 1.0 eq), Cs_2_CO_3_ (3.9 g, 12 mmol, 4 eq), TBAB (0.1 eq), and 18-crown-6 (0.1 eq) were placed in planetary ball mill. The proper alkyl bromide was added, and the reaction was carried out under a nitrogen atmosphere for 1 h. After the completion of the reaction, 30 mL of ethyl acetate was added to the final mixture, and the combined phases were washed with 25 mL of 5% citric acid. The combined aqueous phases were extracted with 3 portions of 25 mL of ethyl acetate. Finally, organic phases were collected, washed with brine, and dried over Na_2_SO_4_. The mixture was filtered, and the solvent was evaporated to yield the crude products as an orange solid, which was purified via column chromatography using silica gel as the stationary phase. 

The spectroscopic data of all synthesized fluoran derivatives are described below:

*3′,6′-dipropoxy-3H-spiro[isobenzofuran-1,9′-xanthen]-3-one* (**1A**), yield – method A: 14%, method B: 23%, yellowish solid, m.p. 75–78 °C, ^1^H NMR (600 MHz, Chloroform-*d*) δ 8.06–7.95 (m), 7.70–7.54 (m), 7.14 (dd, *J* = 7.5, 0.8 Hz), 6.74 (d, *J* = 1.8 Hz), 6.67 (d, *J* = 0.7 Hz), 6.65–6.64 (m), 6.60–6.58 (m), 6.58–6.56 (m), 3.93 (t, *J* = 6.5 Hz), 1.85–1.75 (m), 1.02 (t, *J* = 7.7 Hz) (lit. [62]); ^13^C NMR (151 MHz, Chloroform-*d*) δ 169.62, 161.04, 153.40, 152.68, 135.04, 129.74, 129.16, 127.07, 125.12, 124.09, 112.17, 111.21, 101.50, 83.62, 70.00, 22.57, 10.61; FTIR-ATR [cm^−1^]: 2964.85, 2935.27, 2876.71, 1752.10, 1616.22, 1599.40, 1568.31, 1501.70, 1466.22, 1425.21, 1391.59, 1350.62, 1327.19, 1282.85, 1248.01, 1226.67, 1186.89, 1104.50, 1083.77, 1044.52, 1020.56, 941.75, 900.24, 873.62, 850.00, 825.61, 789.26, 757.62, 731.20, 717.85, 693.77, 673.42, 653.07, 637.97, 617.51, 583.56, 524.37, 465.00, 433.09; HR ESI-MS *m*/*z* calculated for C_26_H_25_O_4_ [M + H]^+^ 417.1697, found [M + H]^+^ 417.1713.

*propyl-2-(3-oxo-6-propoxy-3H-xanthen-9-yl)benzoate* (**1B**), yield – method A: 21%, method B: 53%, method C: 45%, orange solid, m.p. 132–135 °C, lit. 135–137 °C [47], ^1^H NMR (600 MHz, Chloroform-*d*) δ 8.24 (dd, *J* = 7.0, 0.7 Hz), 7.76–7.60 (m), 7.28 (d, *J* = 7.4 Hz), 6.93 (d, *J* = 2.4 Hz), 6.90–6.83 (m), 6.74–6.69 (m), 6.53 (ddd, *J* = 9.7, 1.9, 0.8 Hz), 6.44 (dd, *J* = 1.9, 0.8 Hz), 4.02 (t, *J* = 6.6 Hz), 3.98–3.85 (m), 1.91–1.79 (m), 1.39–1.27 (m), 1.05 (t, *J* = 7.4 Hz), 0.68 (t, *J* = 7.4 Hz) (lit. [47]); ^13^C NMR (151 MHz, Chloroform-*d*) δ 185.61, 165.63, 163.95, 159.09, 154.47, 150.72, 134.32, 132.72, 131.37, 130.94, 130.90, 130.59, 130.44, 129.84, 129.83, 129.15, 117.60, 114.87, 113.95, 105.76, 100.90, 70.54, 67.24, 22.43, 21.70, 10.55, 10.35 (lit. [47]); FTIR-ATR [cm^−1^]: 3058.29, 2966.29, 2937.85, 2877.77, 1723.63, 1644.09, 1589.70, 1542.77, 1515.16, 1465.04, 1417.04, 1377.06, 1348.06, 1251.85, 1210.26, 1167.54, 1140.18, 1120.88, 1104.15,1077.09, 1016.35, 993.76, 959.40, 943.07, 919.30, 901.21, 851.77, 820.53, 803.26, 756.45, 727.92, 708.16, 699.98, 662.80, 639.97, 614.24, 590.12, 571.87, 506.94, 494.05, 455.87, 434.12; HR ESI-MS *m*/*z* calculated for C_26_H_25_O_4_ [M + H]^+^ 417.1697, found [M + H]^+^ 417.1718.

*3′,6′-bis(4-bromobutoxy)-3H-spiro[isobenzofuran-1,9′-xanthen]-3-one* (**2A**), yield – method A: 14%, method B: 23%, yellowish solid, m.p. 82–85 °C, ^1^H NMR (600 MHz, Chloroform-*d*) δ 8.00 (d, *J* = 7.4 Hz, 1H), 7.69–7.57 (m, 2H), 7.14 (d, *J* = 7.5 Hz, 1H), 6.73 (d, *J* = 2.4 Hz, 2H), 6.67 (s, 1H), 6.65 (s, 1H), 6.58 (dd, *J* = 8.8, 2.4 Hz, 2H), 4.01 (t, *J* = 5.9 Hz, 4H), 3.47 (t, *J* = 6.5 Hz, 4H), 2.10–2.00 (m, 4H), 1.99–1.89 (m, 4H); ^13^C NMR (151 MHz, Chloroform-*d*) δ 169.52, 160.70, 153.30, 152.60, 135.08, 131.99, 129.79, 129.22, 126.97, 126.64, 125.13, 124.03, 112.06, 111.47, 101.52, 83.29, 67.34, 33.40, 29.48, 27.85; FTIR-ATR [cm^−1^]: 2951.94, 2936.40, 2899.74, 2871.27, 1755.04, 1715.69, 1611.90, 1569.32, 1505.29, 1467.08, 1426.12, 1397.86, 1361.60,1323.63, 1282.21, 1256.49, 1241.88, 1229.66, 1177.32, 1132.14,1114.54, 1090.41, 1032.21, 1012.61, 958.94, 944.06, 915.21, 870.68, 839.62, 822.92, 799.88, 788.62, 757.99, 715.88, 690.80, 654.03, 638.75, 614.30, 580.48, 550.11, 528.93, 515.70, 462.77, 439.01, 409.92; HR ESI-MS *m*/*z* calculated for C_28_H_27_Br_2_O_5_ [M + H]^+^ 601.0220, found [M + H]^+^ 603.0215;

*4-bromobutyl-2-(6-(4-bromobutoxy)-3-oxo-3H-xanthen-9-yl)benzoate* (**2B**), yield – method A: 20%, method B: 54%, method C: 40%, orange solid, m.p. 59–61 °C, ^1^H NMR (600 MHz, Chloroform-*d*) δ 8.24 (dd, *J* = 7.8, 1.4 Hz, 1H), 7.80–7.59 (m, *J* = 24.3, 7.5, 1.3 Hz, 2H), 7.35–7.27 (m, 1H), 7.04–6.89 (m, 3H), 6.79 (dd, *J* = 9.0, 2.4 Hz, 1H), 6.71–6.62 (m, 2H), 4.13 (t, *J* = 5.8 Hz, 2H), 4.07–3.92 (m, 2H), 3.53–3.41 (m, *J* = 6.0 Hz, 2H), 3.29–3.17 (m, *J* = 10.1, 5.1 Hz, 2H), 2.12–1.90 (m, 4H), 1.66–1.53 (m, 2H), 1.54–1.39 (m, 2H); ^13^C NMR (151 MHz, Chloroform-*d*) δ 184.40, 165.39, 164.14, 159.00, 154.73, 134.03, 132.86, 131.42, 130.51, 130.47, 129.98, 129.32, 129.21, 117.60, 115.10, 114.47, 111.99, 105.58, 101.45, 100.91, 68.13, 64.68, 33.17, 32.81, 29.29, 29.06, 27.64, 27.08; FTIR-ATR [cm^−1^]: 3052.73, 2945.89, 1718.00, 1642.76, 1589.49, 1541.36, 1508.96, 1448.27, 1380.52, 1344.54, 1250.75, 1209.76, 1141.27, 1107.66, 1078.67, 1040.27, 989.64, 919.44, 894.41, 852.96, 757.46, 704.32, 664.16, 612.84, 591.70, 572.93, 531.36, 472.41, 440.60; HR ESI-MS *m*/*z* calculated for C_28_H_27_Br_2_O_5_ [M + H]^+^ 601.0220, found [M + H]^+^ 603.0206.

*3′,6′-bis(hexyloxy)-3H-spiro[isobenzofuran-1,9′-xanthen]-3-one* (**3A**), yield – method A: 12%, method B: 19%, yellowish solid, m.p. 132–135 °C, ^1^H NMR (600 MHz, Chloroform-*d*) δ 8.00 (dd, *J* = 7.4, 1.2 Hz, 1H), 7.69–7.56 (m, 2H), 7.17 (d, *J* = 7.5 Hz, 1H), 6.73 (d, *J* = 2.5 Hz, 2H), 6.65 (s, 1H), 6.63 (s, 1H), 6.56 (d, *J* = 2.4 Hz, 1H), 6.54 (d, *J* = 2.5 Hz, 1H), 4.46–4.26 (m, 2H), 1.59–1.35 (m, 8H), 1.30 (dd, *J* = 6.1, 1.2 Hz, 6H), 0.93 (t, *J* = 7.2 Hz, 6H); ^13^C NMR (151 MHz, Chloroform-*d*) δ 169.61, 161.04, 153.40, 152.67, 135.03, 131.03, 129.73, 129.14, 127.07, 125.11, 124.08, 112.16, 111.19, 101.47, 83.58, 68.52, 31.68, 29.84, 29.19, 25.81, 22.72, 14.16; FTIR-ATR [cm^−1^]: 2934.96, 2855.54, 1747.24, 1632.86, 1614.39, 1571.10, 1503.80, 1464.27, 1421.99, 1393.38, 1350.26, 1279.79, 1246.85, 1193.50, 1106.63, 1024.74, 878.99, 850.00, 835.55, 807.53, 794.65, 766.44, 733.77, 695.06, 672.34, 651.29, 629.24, 587.72, 528.48, 466.74, 430.71; HR ESI-MS *m*/*z* calculated for C_32_H_37_O_5_ [M + H]^+^ 501.2636, found [M + H]^+^ 501.2647.

*hexyl-2-(6-(hexyloxy)-3-oxo-3H-xanthen-9-yl)benzoate* (**3B**), yield – method A: 22%, method B: 55%, method C: 42%, orange solid, m.p. 110–112 °C, lit. 112–113 °C [48], ^1^H NMR (600 MHz, Chloroform-*d*) δ 8.23 (dt, *J* = 7.9, 2.1 Hz, 1H), 7.74–7.59 (m, 2H), 7.35–7.20 (m, 1H), 6.96–6.80 (m, 3H), 6.73–6.64 (m, 1H), 6.57–6.48 (m, 1H), 6.45 (s, 1H), 4.79 (h, *J* = 6.2 Hz, 1H), 4.48 (h, *J* = 6.2 Hz, 1H), 1.80–1.38 (m, 4H), 1.34 (dd, *J* = 6.0, 1.6 Hz, 3H), 1.22–1.00 (m, 4H), 0.93 (t, *J* = 7.3 Hz, 3H), 0.86 (d, *J* = 6.1 Hz, 3H), 0.76–0.58 (m, 3H) (lit. [48]); ^13^C NMR (151 MHz, Chloroform-*d*) δ 185.28, 165.43, 163.70, 158.79, 154.20, 150.43, 133.97, 132.43, 131.15, 130.67, 130.32, 130.17, 129.58, 129.56, 128.91, 117.32, 114.61, 113.69, 105.50, 100.60, 68.84, 65.56, 31.34, 31.20, 28.75, 28.07, 25.46, 25.34, 22.41, 22.24, 13.88, 13.85 (lit. [48]); FTIR-ATR [cm^−1^]: 3050.29, 2931.23, 2856.75, 1712.32, 1641.59, 1592.54, 1571.46, 1545.51, 1520.14, 1466.64, 1442.77, 1420.61, 1374.31, 1339.65, 1280.76, 1251.63, 1209.21, 1119.74, 1104.85, 1041.72, 1019.08, 1000.70, 974.13, 918.51, 902.14, 874.23, 859.50, 822.34, 800.50, 760.77, 715.17, 698.47, 665.66, 657.61, 615.37, 587.54, 570.18, 547.06, 523.39, 503.89, 481.36, 442.60, 427.58; HR ESI-MS *m*/*z* calculated for C_32_H_37_O_5_ [M + H]^+^ 501.2636, found [M + H]^+^ 501.2661.

*3′,6′-bis(heptyloxy)-3H-spiro[isobenzofuran-1,9′-xanthen]-3-one* (**4A**), yield – method A: 7%, method B: 18%, yellowish solid, m.p. 66–69 °C, ^1^H NMR (600 MHz, Chloroform-*d*) δ 8.00 (dd, *J* = 7.0, 1.4 Hz, 1H), 7.70–7.55 (m, 2H), 7.14 (d, *J* = 7.5 Hz, 1H), 6.74 (d, *J* = 2.4 Hz, 2H), 6.66 (s, 1H), 6.64 (s, 1H), 6.59 (d, *J* = 2.5 Hz, 1H), 6.57 (d, *J* = 2.4 Hz, 1H), 3.96 (t, *J* = 6.6 Hz, 4H), 1.84–1.71 (m, 4H), 1.66 – 1.48 (m, 3H), 1.33 (dt, *J* = 7.6, 3.5 Hz, 6H), 0.95–0.80 (m, 9H); ^13^C NMR (151 MHz, Chloroform-*d*) δ 169.62, 161.04, 153.41, 152.68, 135.03, 129.73, 129.15, 127.08, 125.12, 124.09, 112.18, 111.19, 101.48, 68.53, 31.91, 29.23, 29.16, 26.11, 22.75, 14.22; FTIR-ATR [cm^−1^]: 2931.48, 2869.66, 2854.15, 1753.95, 1631.55, 1610.94, 1572.12, 1502.82, 1475.36, 1463.95, 1423.45, 1393.02, 1346.88, 1279.06, 1247.59, 1223.11, 1192.90, 1167.75, 1105.00, 1086.57, 1038.12, 1013.31, 961.77, 880.29, 847.10, 831.32, 795.31, 764.56, 726.11, 697.66, 677.75, 650.24, 629.76, 586.14, 528.13, 467.46; HR ESI-MS *m*/*z* calculated for C_34_H_41_O_5_ [M + H]^+^ 529.2949, found [M + H]^+^ 529.2957.

*heptyl-2-(6-(heptyloxy)-3-oxo-3H-xanthen-9-yl)benzoate* (**4B**), yield – method A: 12%, method B: 50%, method C: 39%, orange solid, m.p. 59–62 °C, ^1^H NMR (600 MHz, Chloroform-*d*) δ 8.23 (dd, *J* = 8.4, 2.9 Hz, 1H), 7.81–7.54 (m, 2H), 7.39–7.17 (m, 1H), 7.02–6.80 (m, 3H), 6.77–6.66 (m, 1H), 6.61–6.50 (m, 1H), 6.46–6.40 (m, 1H), 4.22–3.82 (m, 5H), 1.98–1.67 (m, 4H), 1.63–1.21 (m, 10H), 1.21–0.95 (m, 3H), 0.94–0.76 (m, 4H); ^13^C NMR (151 MHz, Chloroform-d) δ 185.28, 165.69, 164.06, 159.06, 154.59, 134.24, 132.62, 131.41, 130.94, 130.54, 130.43, 129.78, 129.71, 129.14, 117.63, 114.92, 114.14, 105.76, 100.78, 69.13, 65.88, 31.82, 31.65, 29.07, 29.03, 28.91, 28.33, 26.00, 25.85, 22.68, 22.65, 14.16, 14.14; FTIR-ATR [cm^−1^]: 3039.34, 2952.88, 2920.79, 2852.33, 1712.42, 1643.40, 1594.91, 1570.48, 1548.06, 1518.21, 1485.02, 1445.24, 1421.52, 1377.68, 1342.53, 1282.19, 1254.58, 1212.14, 1132.31, 1105.06, 1031.23, 1010.81, 994.78, 974.96, 928.54, 902.94, 882.74, 860.27, 822.75, 801.68, 760.90, 729.64, 703.49, 664.74, 625.46, 612.91, 590.32, 571.92, 530.67, 507.02, 482.60, 466.49, 442.08, 405.50; HR ESI-MS *m*/*z* calculated for C_34_H_41_O_5_ [M + H]^+^ 529.2949, found [M + H]^+^ 529.2977.

*3′,6′-bis(pentan-2-yloxy)-3H-spiro[isobenzofuran-1,9′-xanthen]-3-one* (**5A**), yield – method A: 8%, method B: 15%, yellowish solid, m.p. 180–182 °C, ^1^H NMR (600 MHz, Chloroform-*d*) δ 8.00 (dd, *J* = 7.0, 1.4 Hz, 1H), 7.62 (dtd, *J* = 20.5, 7.4, 1.2 Hz, 2H), 7.14 (d, *J* = 7.6 Hz, 1H), 6.74 (d, *J* = 2.4 Hz, 2H), 6.66 (s, 1H), 6.64 (s, 1H), 6.59 (d, *J* = 2.4 Hz, 1H), 6.57 (d, *J* = 2.4 Hz, 1H), 4.11 (t, *J* = 6.8 Hz, 2H), 3.96 (t, *J* = 6.6 Hz, 2H), 1.85–1.73 (m, 4H), 1.72–1.57 (m, 4H), 1.51–1.20 (m, 12H), 1.07–0.72 (m, 6H); ^13^C NMR (151 MHz, Chloroform-*d*) δ 169.58, 160.20, 153.27, 152.75, 134.99, 129.72, 129.22, 127.19, 125.10, 124.17, 113.06, 113.00, 111.08, 102.54, 102.49, 83.68, 74.17, 38.64, 38.62, 29.85, 19.78, 19.76, 18.88, 18.86, 14.15; FTIR-ATR [cm^-1^]: 2966.11, 2936.91, 2877.01, 1750.74, 1616.51, 1568.30, 1501.28, 1466.22, 1425.11, 1391.86, 1348.66, 1326.02, 1283.04, 1247.83, 1227.01, 1186.81, 1105.06, 1083.81, 1044.65, 1020.70, 970.09, 942.03, 901.77, 873.95, 850.05, 825.83, 789.08, 757.25, 730.85, 717.25, 693.85, 674.88, 652.72, 637.62, 617.26, 583.19, 523.67, 464.96, 432.87; HR ESI-MS *m*/*z* calculated for C_30_H_33_O_5_ [M + H]^+^ 473.2323, found [M + H]^+^ 473.2338.

*pentan-2-yl-2-(3-oxo-6-(pentan-2-yloxy)-3H-xanthen-9-yl)benzoate* (**5B**), yield – method A: 16%, method B: 31%, method C: 25%, orange solid, m.p. 105–108 °C, ^1^H NMR (600 MHz, Chloroform-*d*) δ 8.23 (dd, *J* = 7.7, 1.4 Hz, 1H), 7.77–7.56 (m, 2H), 7.35–7.21 (m, 1H), 6.91 (d, *J* = 2.9 Hz, 1H), 6.89–6.82 (m, 2H), 6.71 (ddd, *J* = 8.9, 2.7, 0.9 Hz, 1H), 6.52 (dt, *J* = 9.8, 1.5 Hz, 1H), 6.43 (t, *J* = 1.5 Hz, 1H), 4.04 (t, *J* = 6.5 Hz, 2H), 3.94 (t, *J* = 6.6, 1.4 Hz, 2H), 1.90–1.78 (m, 2H), 1.51–1.41 (m, 2H), 1.40–1.16 (m, 10H), 1.16–1.06 (m, 4H), 1.05–0.93 (m, 2H), 0.91–0.81 (m, 6H); ^13^C NMR (151 MHz, Chloroform-*d*) δ 185.45, 165.16, 163.24, 159.00, 154.50, 150.92, 133.93, 132.43, 131.35, 130.47, 130.28, 129.63, 129.39, 129.24, 117.54, 117.48, 114.83, 114.57, 105.62, 101.50, 74.84, 72.30, 38.27, 37.79, 19.52, 19.20, 18.43, 18.33, 13.96, 13.75; FTIR-ATR [cm^−1^]: 3055.08 2961.44, 2931.80, 2871.93,1708.62, 1643.67, 1592.37, 1546.42, 1515.59, 1478.44, 1445.98, 1418.99, 1377.12, 1343.51, 1279.95, 1251.73, 1206.85, 1101.63, 1057.18, 1041.56, 988.71, 936.47, 919.05, 885.12, 850.91, 827.10, 759.23, 709.33, 664.66, 612.23, 590.54, 570.93, 498.99, 442.90; HR ESI-MS *m*/*z* calculated for C_30_H_33_O_5_ [M + H]^+^ 473.2323, found [M + H]^+^ 473.2346.

## 4. Conclusions

In summary, this paper presents the synthesis of 10 fluoran derivatives substituted with different length alkyl chains. As a result, we also present six novel xanthene dyes, which spectroscopic and chemical properties were examined. Syntheses with classical heating as well as with the microwave-assisted method allowed us to obtain two different luminescent and leuco dyes, with closed (**A**) and open (**B**) spironolactone rings simultaneously during one-step synthesis. The use of a microwave reactor allowed us to reduce the reaction time from 24 h to only 10 min with higher yields, up to 77%. With the use of the eco-friendly ball mill method, it was only possible to obtain one compound, **B**, which facilitated its separation and purification. We observed different spectroscopic behaviors for all of the compounds compared to xanthene dyes; their luminescence intensity increased with the increasing solution concentration. Furthermore, the relationship between the length of the alkyl chain and the time of luminescence decay was investigated, although the specific emission mechanism should be more widely examined. We are the first to present this combination of an ionic liquid as a developer and a synthesized closed form of fluoran derivative **A** after heating changed the color irreversibly, which can be used in various types of overheating sensors.

## Data Availability

Data are contained within the supplementary material.

## Supplementary Materials

The following supporting information can be downloaded at: https://www.mdpi.com/article/10.3390/molecules27103092/s1, Section S1: Spectroscopic characterization (Figure S1. ^1^H-NMR (600 MHz, chloroform-*d*) spectrum of product **1A**, Figure S2. ^13^C-NMR (151 MHz, chloroform-*d*) spectrum of product **1A,** Figure S3. FTIR-ATR spectrum of product **1A**, Figure S4. HR ESI-MS spectrum of product **1A**, Figure S5. ^1^H-NMR (600 MHz, chloroform-*d*) spectrum of product **1B**, Figure S6. ^13^C-NMR (151 MHz, chloroform-*d*) spectrum of product **1B**, Figure S7. FTIR-ATR spectrum of product **1B**, Figure S8. HR ESI-MS spectrum of product **1B,** Figure S9. ^1^H-NMR (600 MHz, chloroform-*d*) spectrum of product **2A**, Figure S10. ^13^C-NMR (151 MHz, chloroform-*d*) spectrum of product **2A**, Figure S11. FTIR-ATR spectrum of product **2A,** Figure S12. HR ESI-MS spectrum of product **2A**, Figure S13. ^1^H-NMR (600 MHz, chloroform-*d*) spectrum of product **2B**, Figure S14. ^13^C-NMR (151 MHz, chloroform-*d*) spectrum of product **2B**, Figure S15. FTIR-ATR spectrum of product **2B,** Figure S16. HR ESI-MS spectrum of product **2B,** Figure S17. ^1^H-NMR (600 MHz, chloroform-*d*) spectrum of product **3A**, Figure S18. ^13^C-NMR (151 MHz, chloroform-*d*) spectrum of product **3A**, Figure S19. FTIR-ATR spectrum of product **3A**, Figure S20. HR ESI-MS spectrum of product **3A**, Figure S21. ^1^H-NMR (600 MHz, chloroform-*d*) spectrum of product **3B**, Figure S22. ^13^C-NMR (151 MHz, chloroform-*d*) spectrum of product **3B**, Figure S23. FTIR-ATR spectrum of product **3B**, Figure S24. HR ESI-MS spectrum of product **3B**, Figure S25. ^1^H-NMR (600 MHz, chloroform-*d*) spectrum of product **4A**, Figure S26. ^13^C-NMR (151 MHz, chloroform-*d*) spectrum of product **4A**, Figure S27. FTIR-ATR spectrum of product **4A**, Figure S28. HR ESI-MS spectrum of product **4A**, Figure S29. ^1^H-NMR (600 MHz, chloroform-*d*) spectrum of product **4B**, Figure S30. ^13^C-NMR (151 MHz, chloroform-*d*) spectrum of product **4B**, Figure S31. FTIR-ATR spectrum of product **4B**, Figure S32. HR ESI-MS spectrum of product **4B**, Figure S33. ^1^H-NMR (600 MHz, chloroform-*d*) spectrum of product **5A**, Figure S34. ^13^C-NMR (151 MHz, chloroform-*d*) spectrum of product **5A**, Figure S35. FTIR-ATR spectrum of product **5A**, Figure S36. HR ESI-MS spectrum of product **5A**, Figure S37. ^1^H-NMR (600 MHz, chloroform-*d*) spectrum of product **5B**, Figure S38. ^13^C-NMR (151 MHz, chloroform-*d*) spectrum of product **5B**, Figure S39. FTIR-ATR spectrum of product **5B**, Figure S40. HR ESI-MS spectrum of product **5B**); Section S2: Spectroscopic properties—Table S1: Summary of spectroscopic properties measured for all compounds (Maximum absorption, emission, fluorescence lifetimes, quantum yields); Figure S41: Emission spectra for different concentrations measured for synthesized compounds (**a**) **1B**, (**b**) **2B**, (**c**) **5B**, (**d**) **3A**, (**e**) **5A**.

## Sample Availability

Samples of the compounds are not available from the authors.
