# Supplementary material for: Controlled Synthesis of Luminescent Xanthene Dyes and Use of Ionic Liquid in Thermochromic Reaction
Source: Molecules. 2022 May 11;27(10):3092. doi: 10.3390/molecules27103092 (PMC9143205; doi:10.3390/molecules27103092)
Supplement: Supplementary file 1 [file molecules-27-03092-s001.zip › molecules-1669927-supplementary.pdf]

Supplementary Information

Controlled Synthesis of Luminescent Xanthene Dyes and Use of Ionic Liquid in Thermochromic Reaction

Bartłomiej Potaniec[1,†], Maria Zdończyk[1,2,†] and Joanna Cybińska[1,2,*]

[1] Advanced Materials Synthesis Group, Materials Science & Engineering Center, Łukasiewicz Research Network—PORT Polish Center for Technology Development, Stabłowicka 147 Street, 54-066 Wrocław, Poland; bartlomiej.potaniec@port.lukasiewicz.gov.pl;

[2] Faculty of Chemistry, University of Wroclaw, F. Joliot-Curie 14 Street, 50-383 Wrocław, Poland; maria.zdonczyk@chem.uni.wroc.pl

***** Correspondence: joanna.cybinska@chem.uni.wroc.pl or joanna.cybinska@port.lukasiewicz.gov.pl

[†] These authors contributed equally to this work.

S1. Spectroscopic characterization

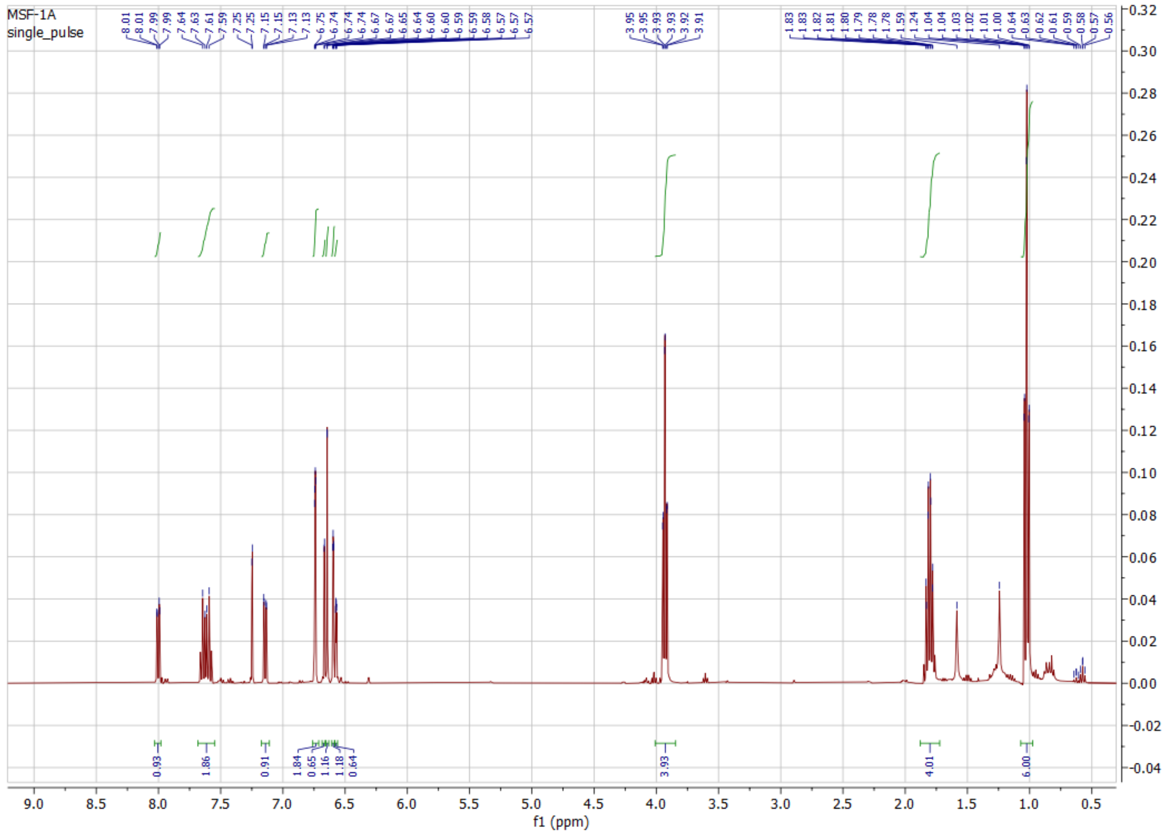

Figure S1. ^{1}H-NMR (600 MHz, chloroform-*d*) spectrum of product **1A**.

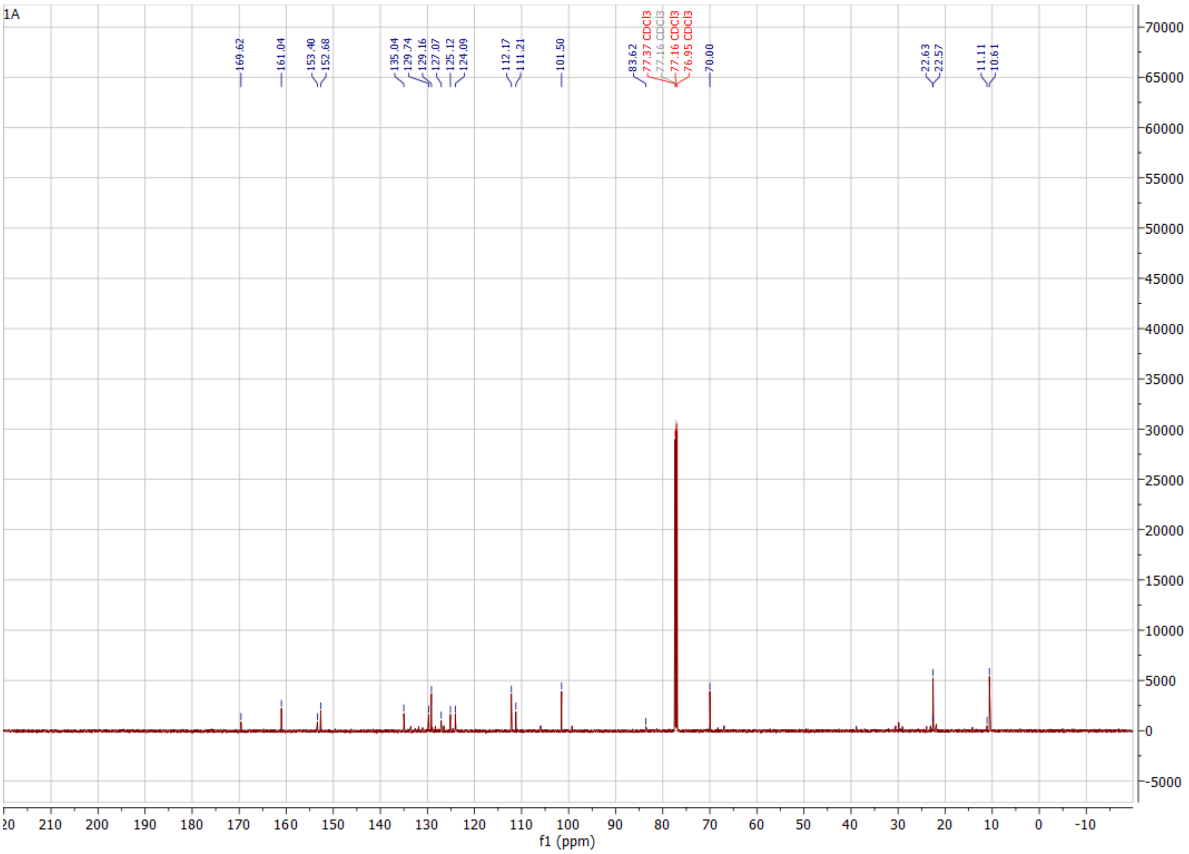

Figure S2. ^{13}C-NMR (151 MHz, chloroform-*d*) spectrum of product **1A**.

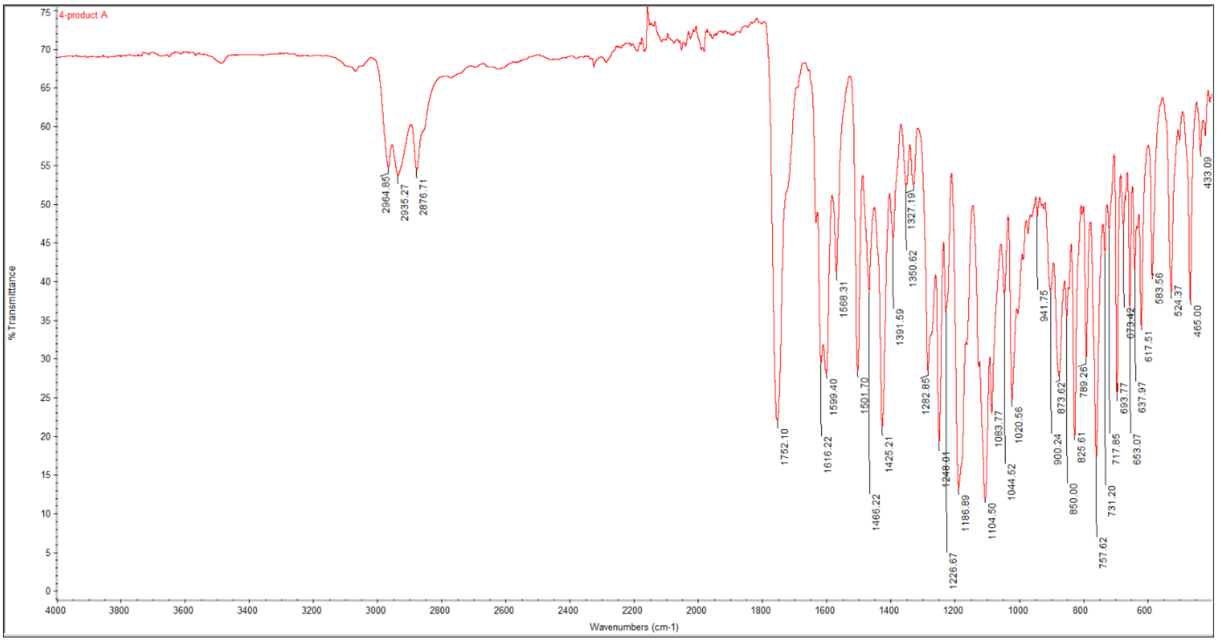

Figure S3. FTIR-ATR spectrum of product **1A**.

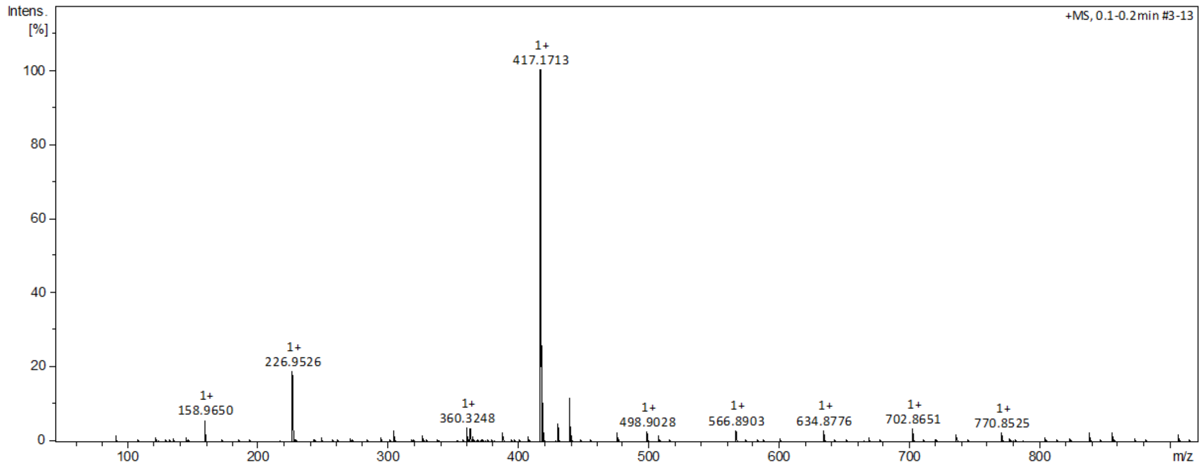

Figure S4. HR ESI-MS spectrum of product **1A**.

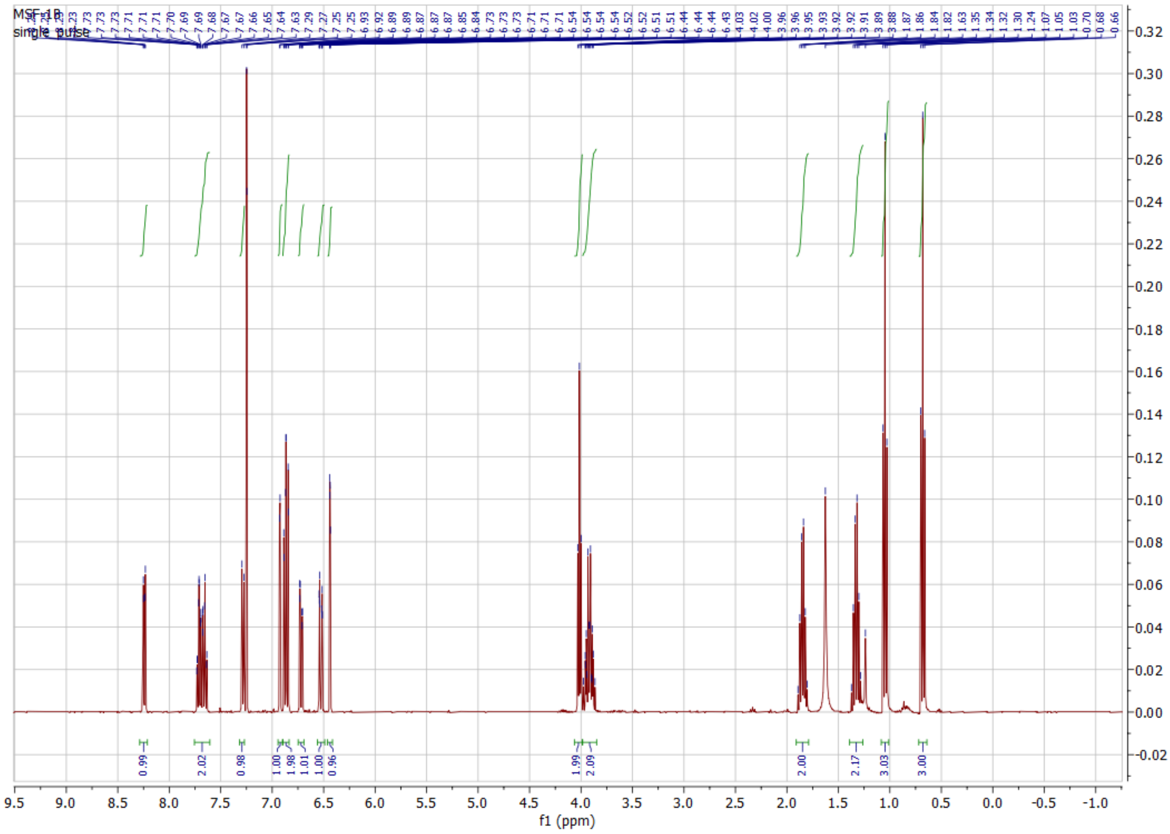

Figure S5. ^{1}H-NMR (600 MHz, chloroform-*d*) spectrum of product **1B**.

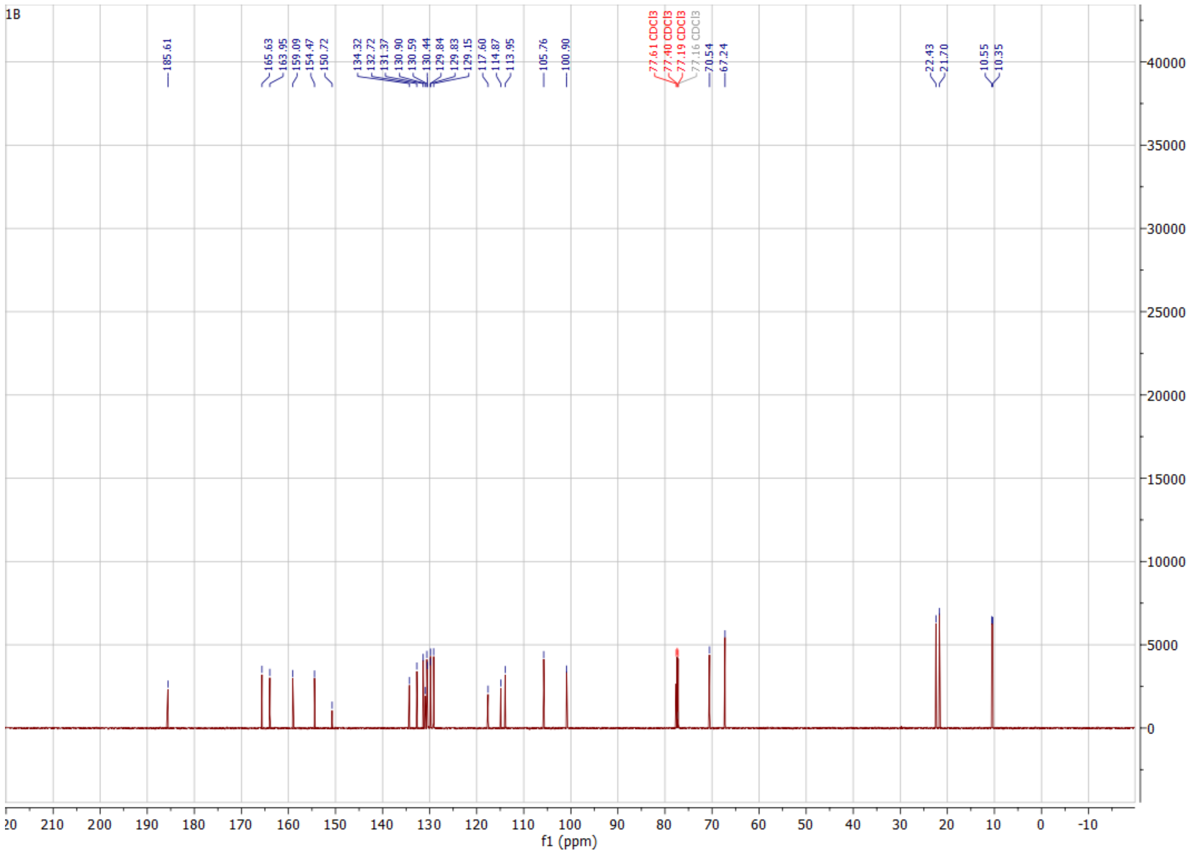

Figure S6. ^{13}C-NMR (151 MHz, chloroform-*d*) spectrum of product **1B**.

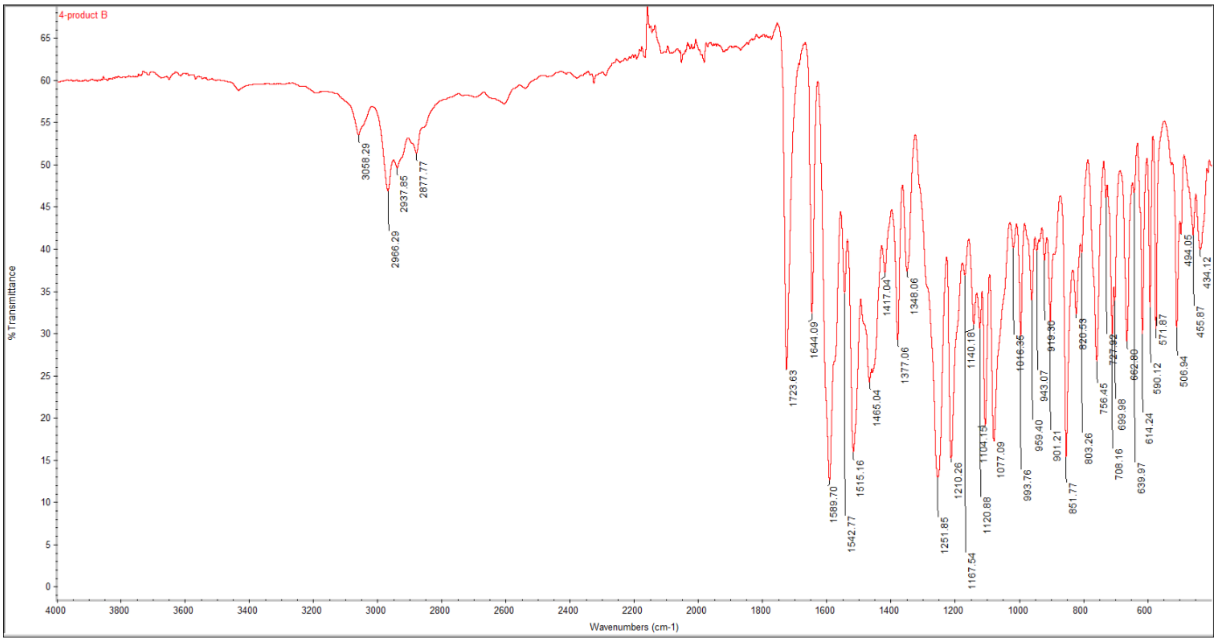

Figure S7. FTIR-ATR spectrum of product **1B**.

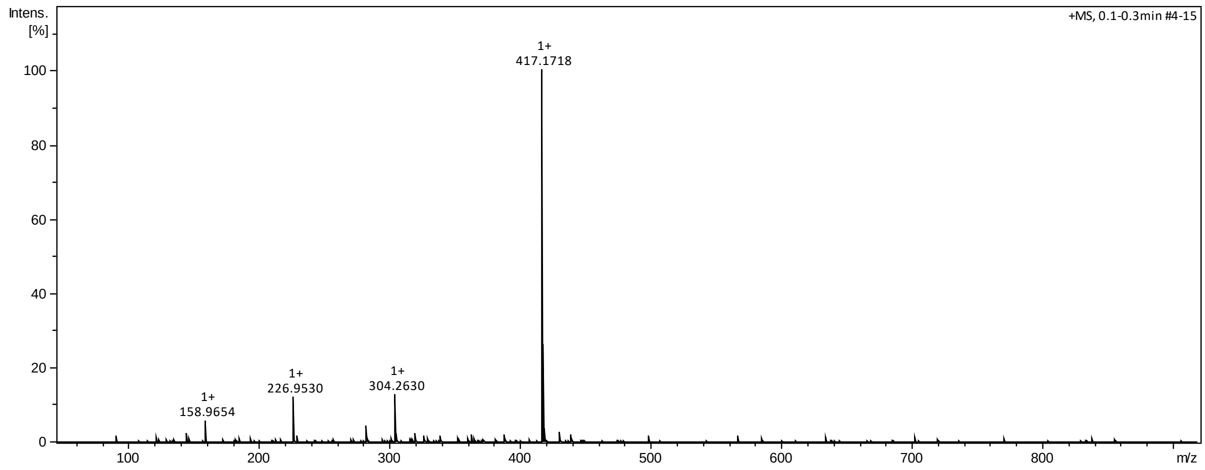

Figure S8. HR ESI-MS spectrum of product **1B**.

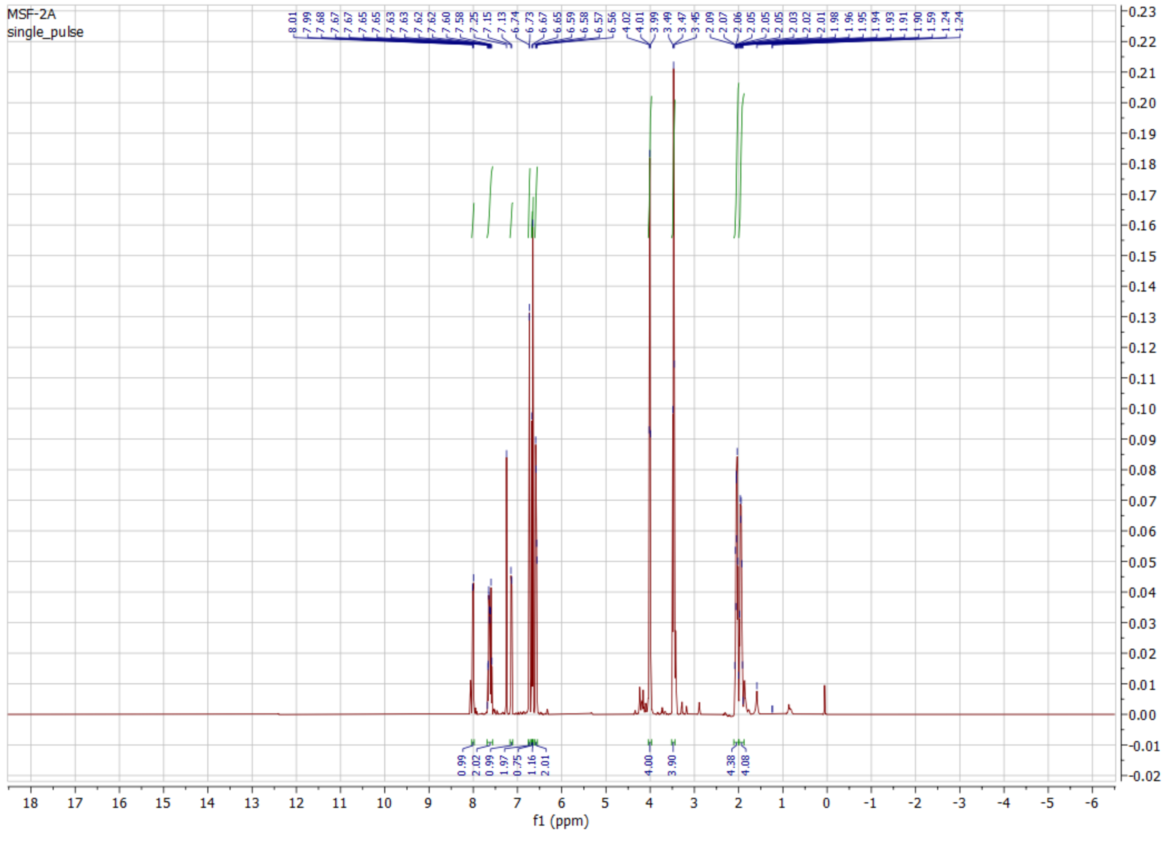

Figure S9. ^{1}H-NMR (600 MHz, chloroform-*d*) spectrum of product **2A**.

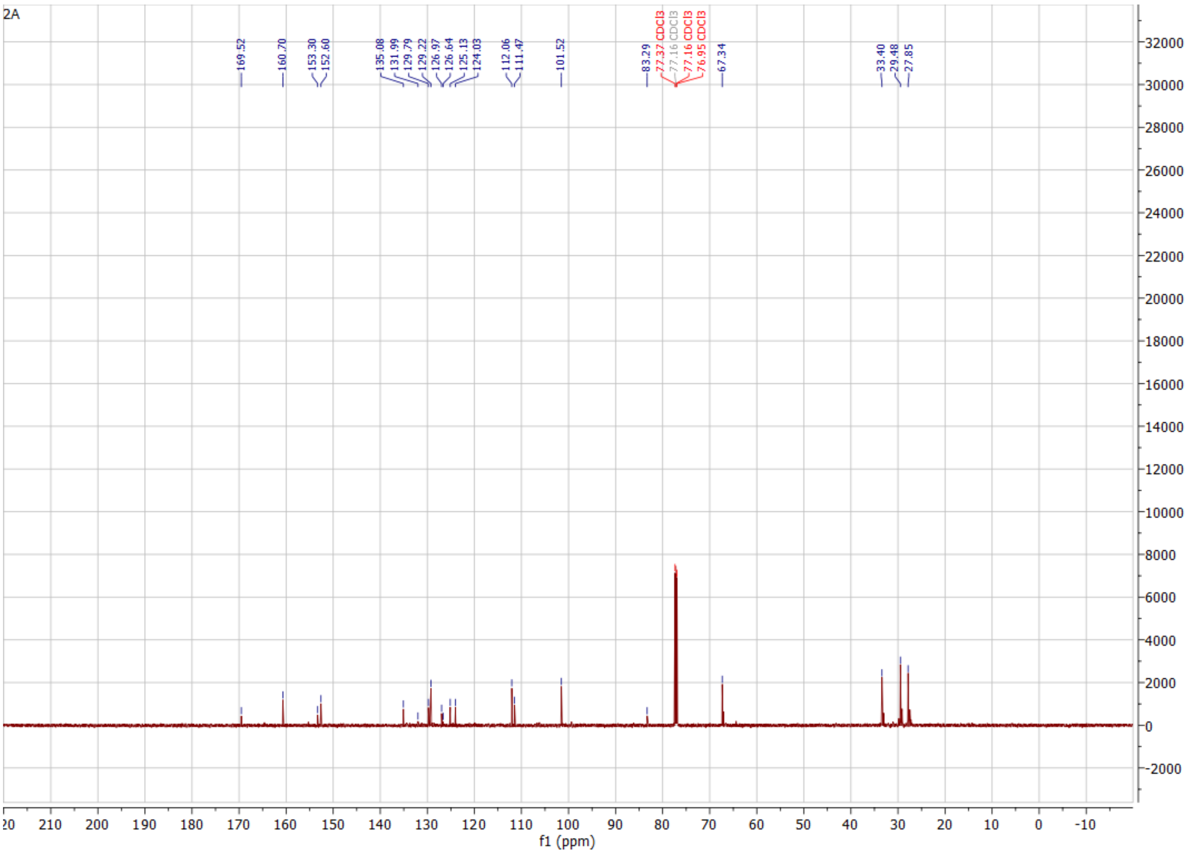

Figure S10. ^{13}C-NMR (151 MHz, chloroform-*d*) spectrum of product **2A**.

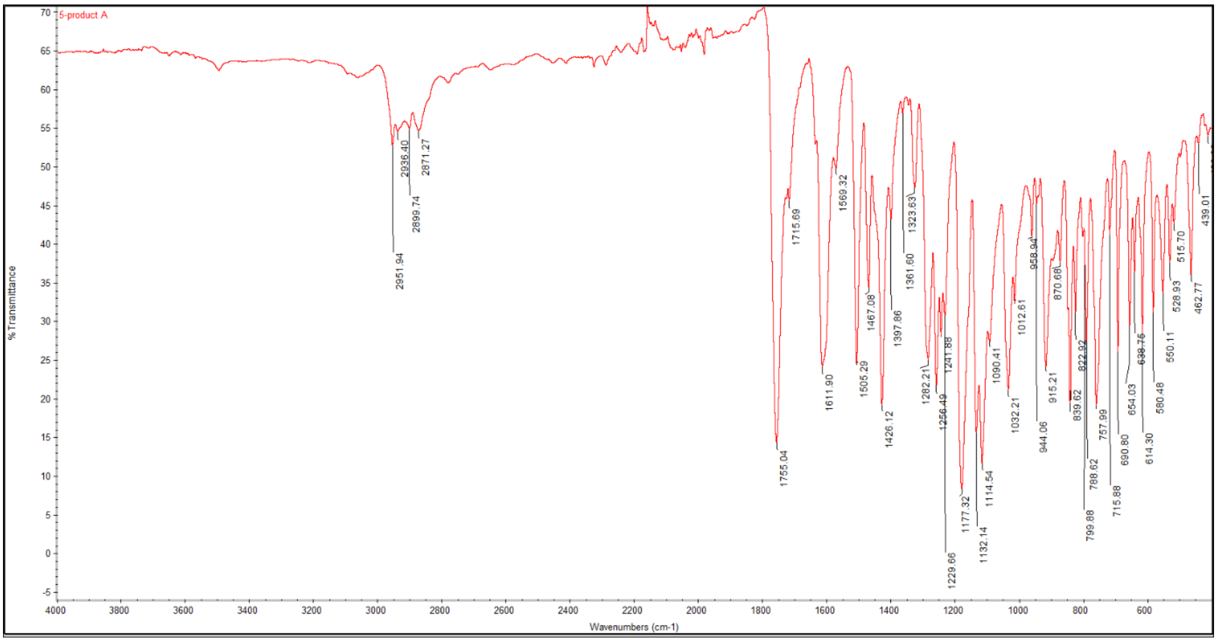

Figure S11. FTIR-ATR spectrum of product **2A**.

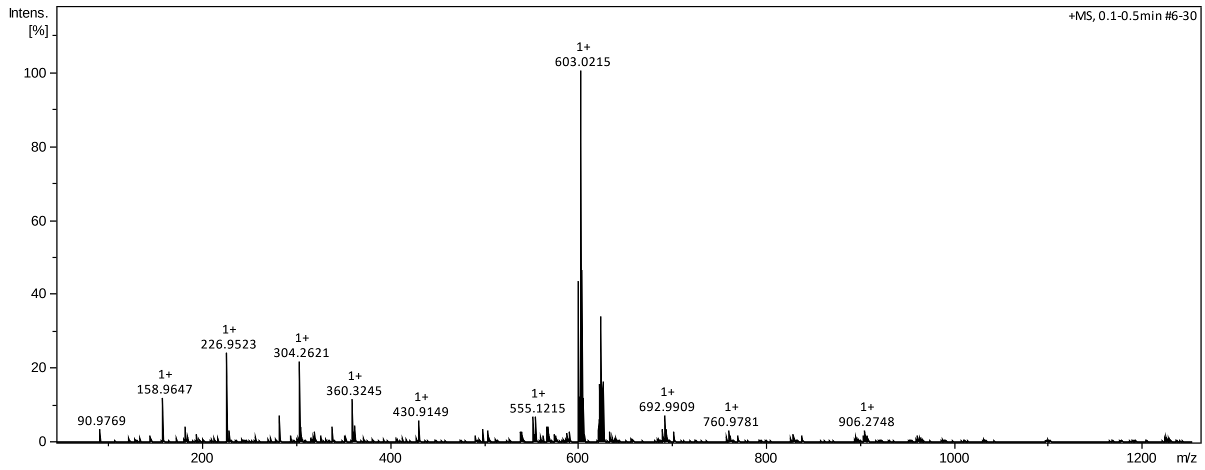

Figure S12. HR ESI-MS spectrum of product **2A**.

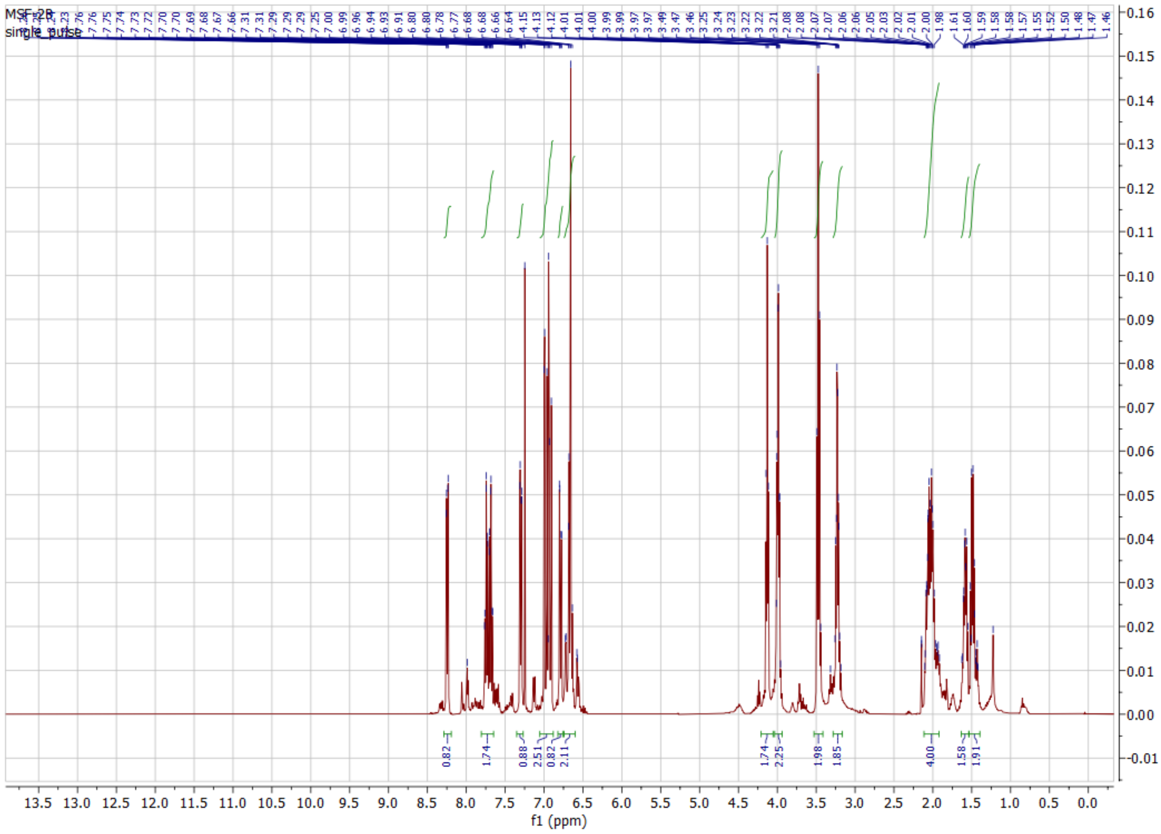

Figure S13. ^{1}H-NMR (600 MHz, chloroform-*d*) spectrum of product **2B**.

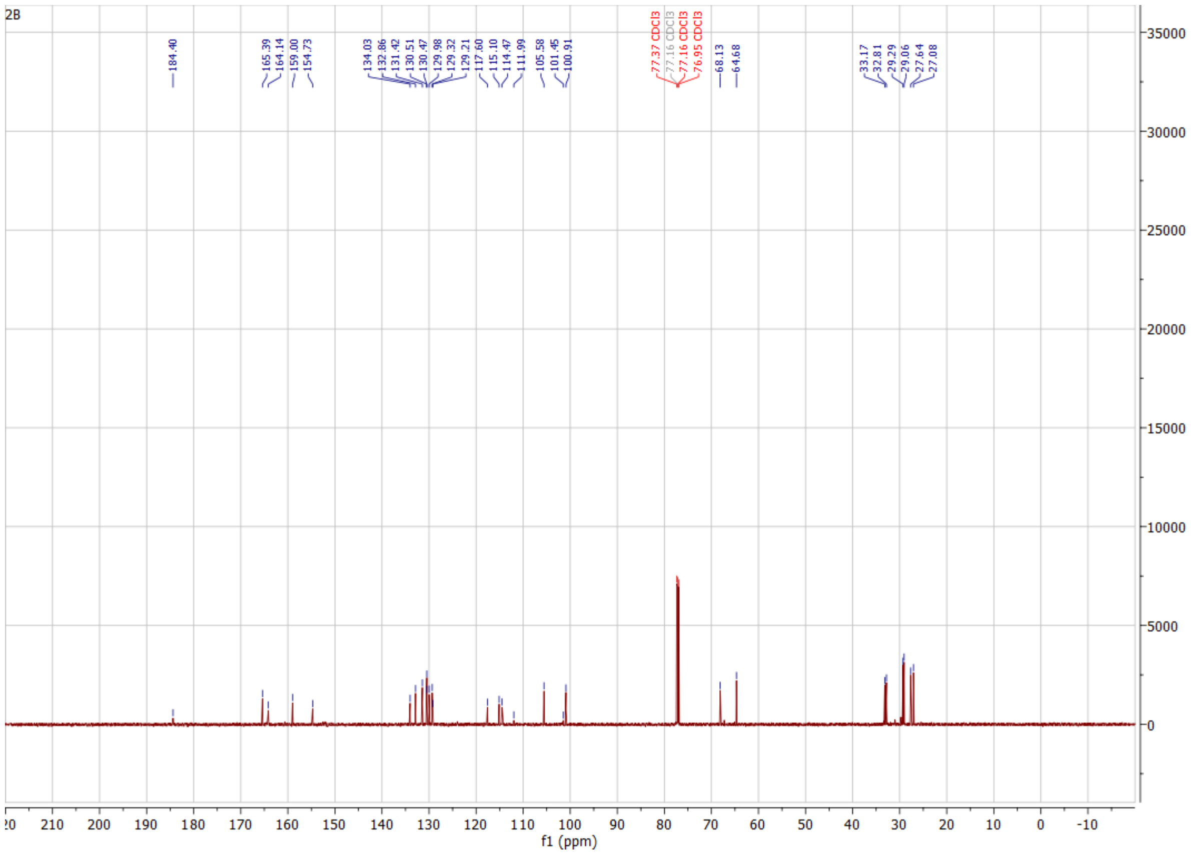

Figure S14. ^{13}C-NMR (151 MHz, chloroform-*d*) spectrum of product **2B**.

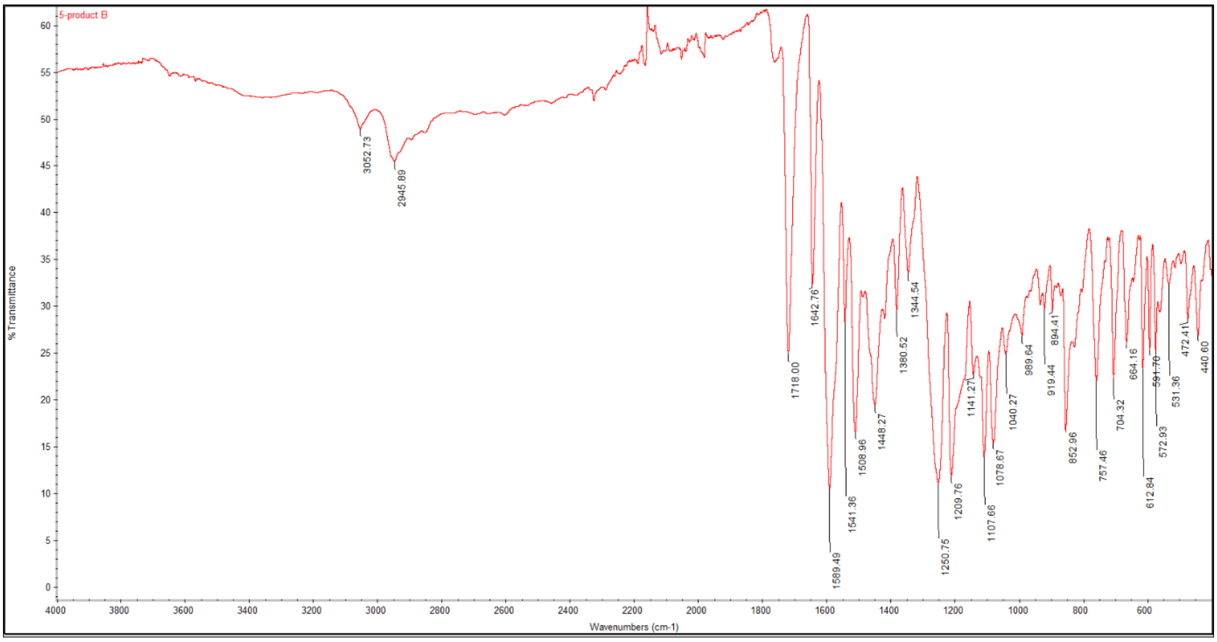

Figure S15. FTIR-ATR spectrum of product **2B**.

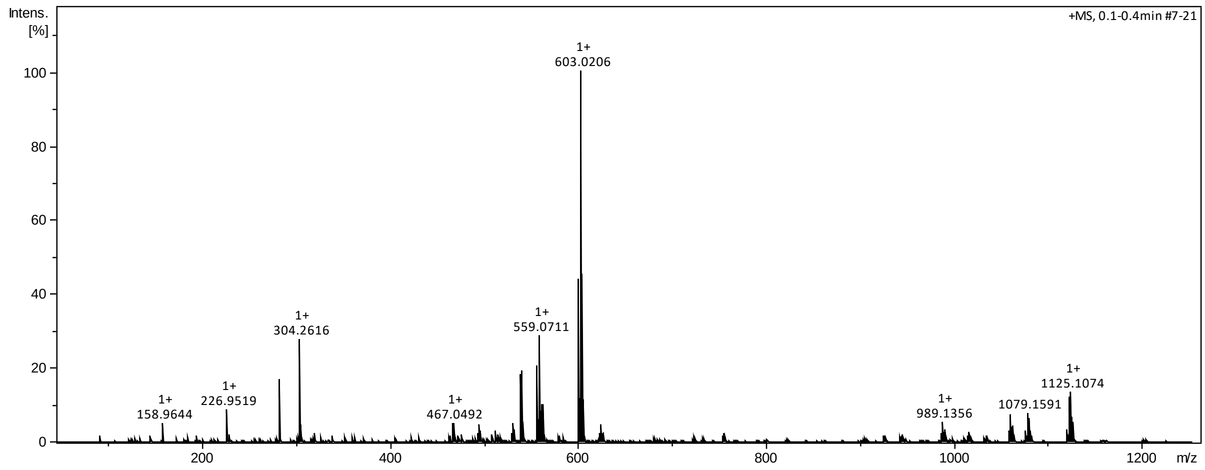

Figure S16. HR ESI-MS spectrum of product **2B**.

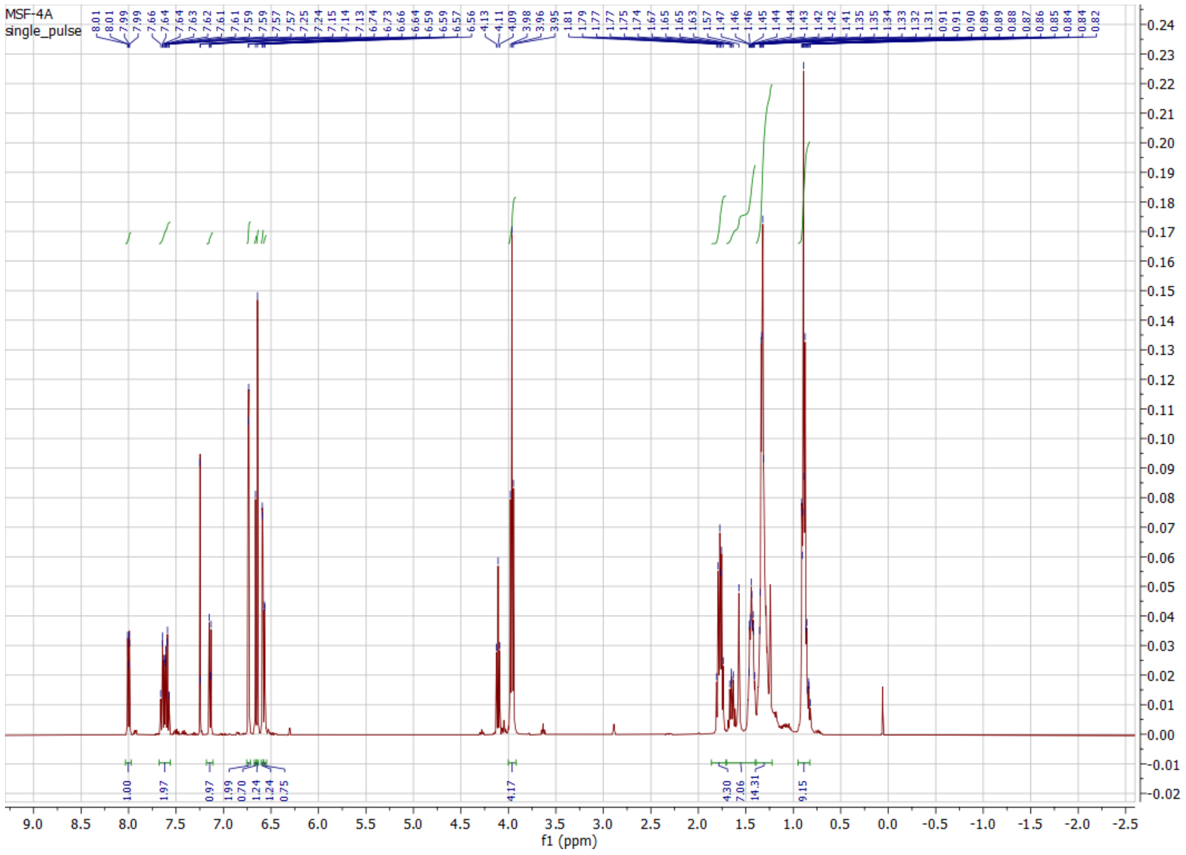

Figure S17. ^{1}H-NMR (600 MHz, chloroform-*d*) spectrum of product **3A**.

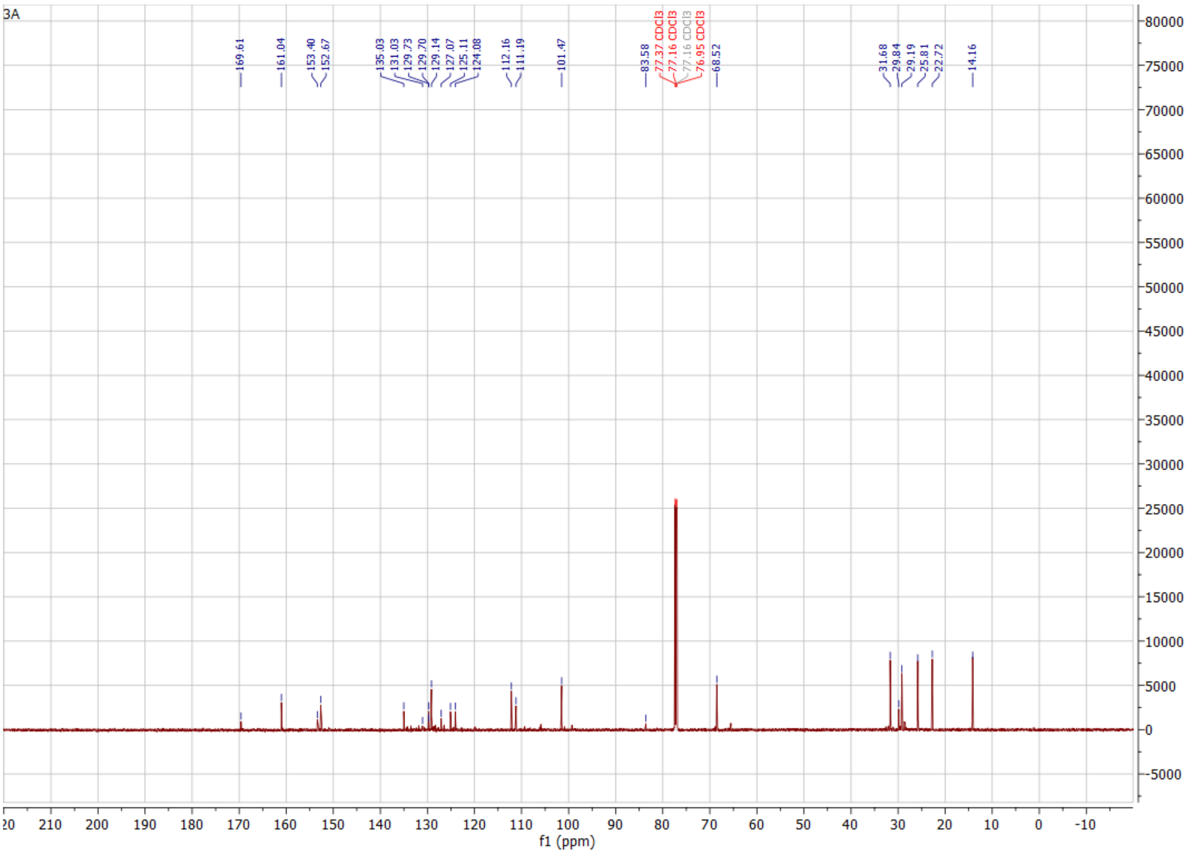

Figure S18. ^{13}C-NMR (151 MHz, chloroform-*d*) spectrum of product **3A**.

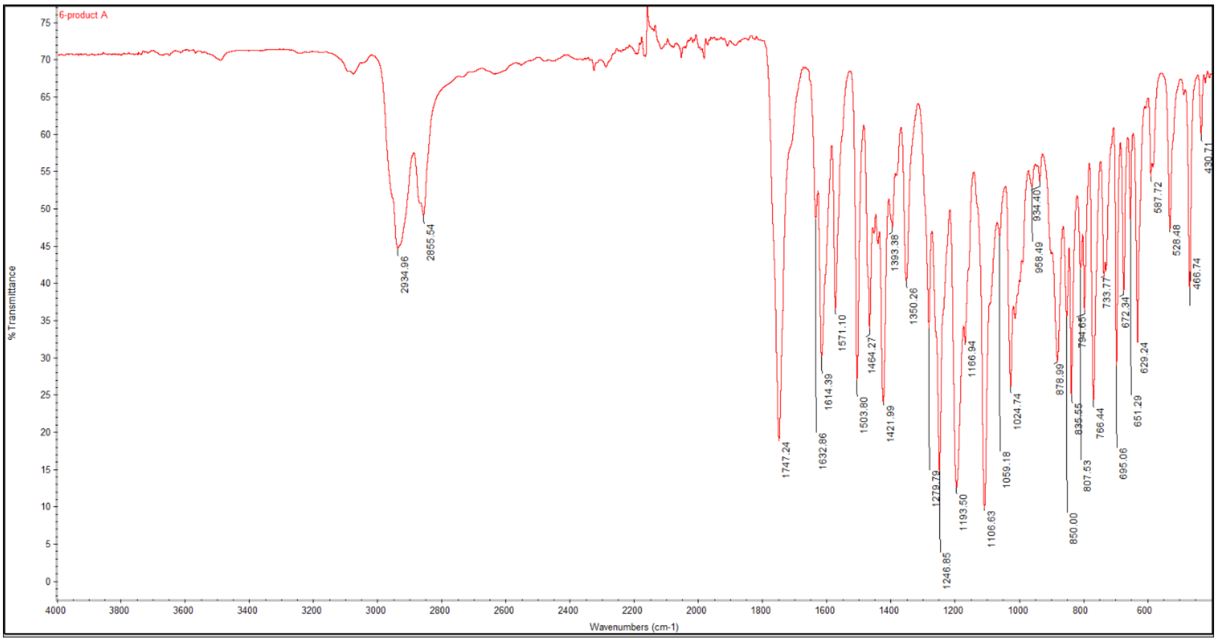

Figure S19. FTIR-ATR spectrum of product **3A**.

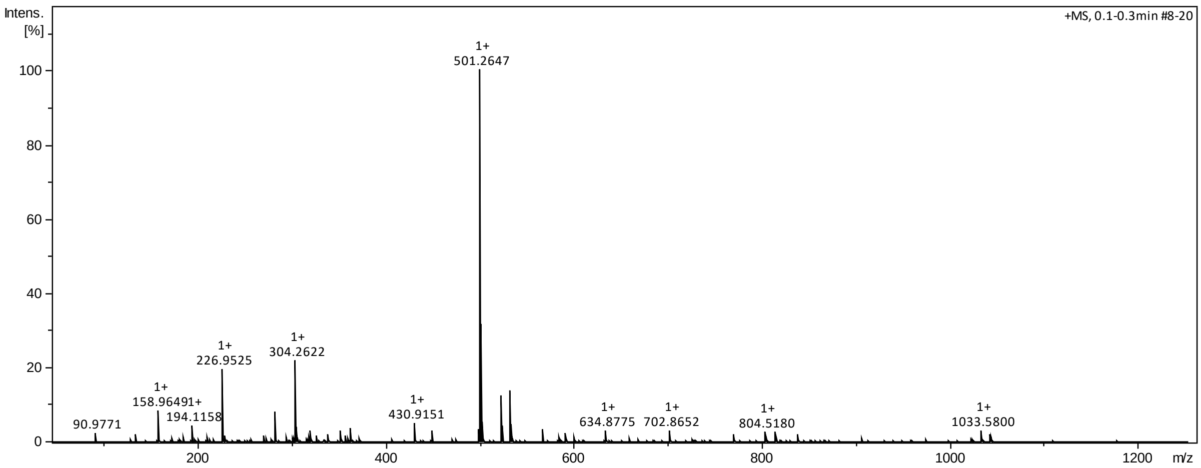

Figure S20. HR ESI-MS spectrum of product **3A**.

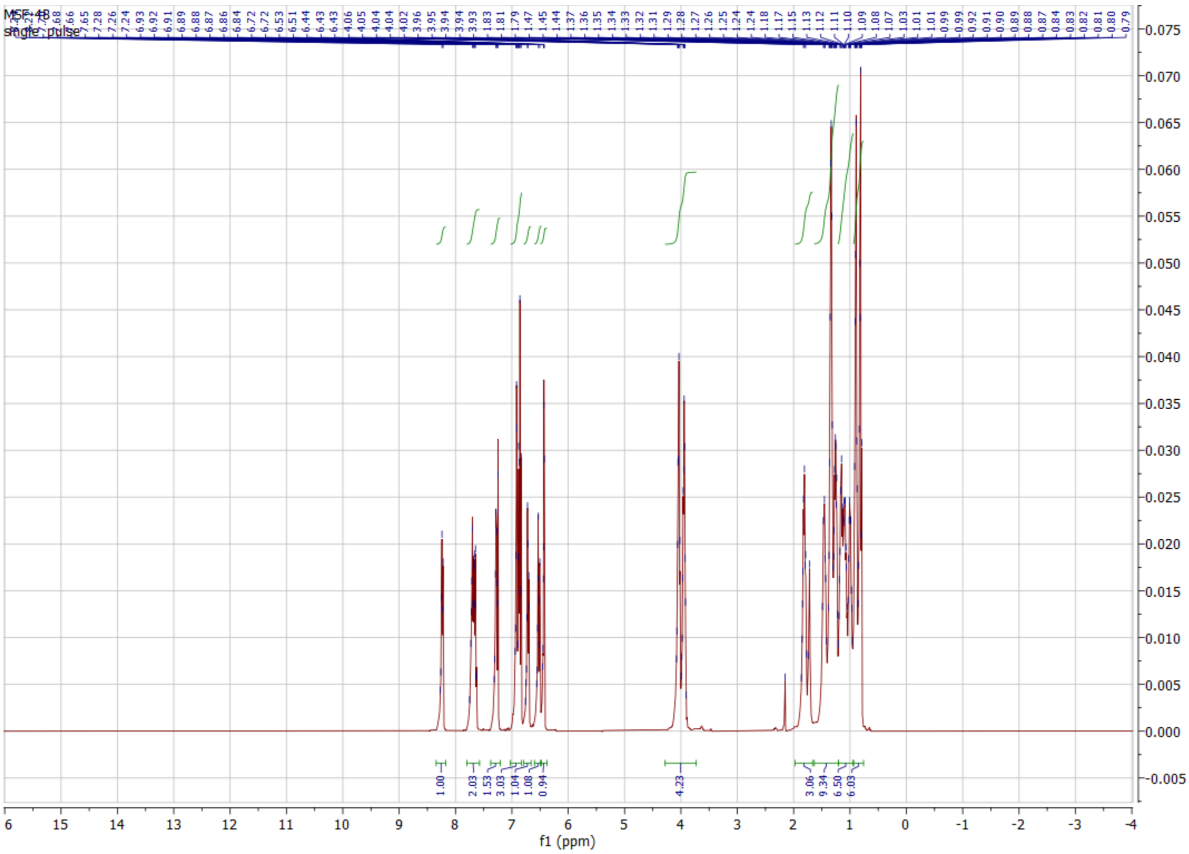

Figure S21. ^{1}H-NMR (600 MHz, chloroform-*d*) spectrum of product **3B**.

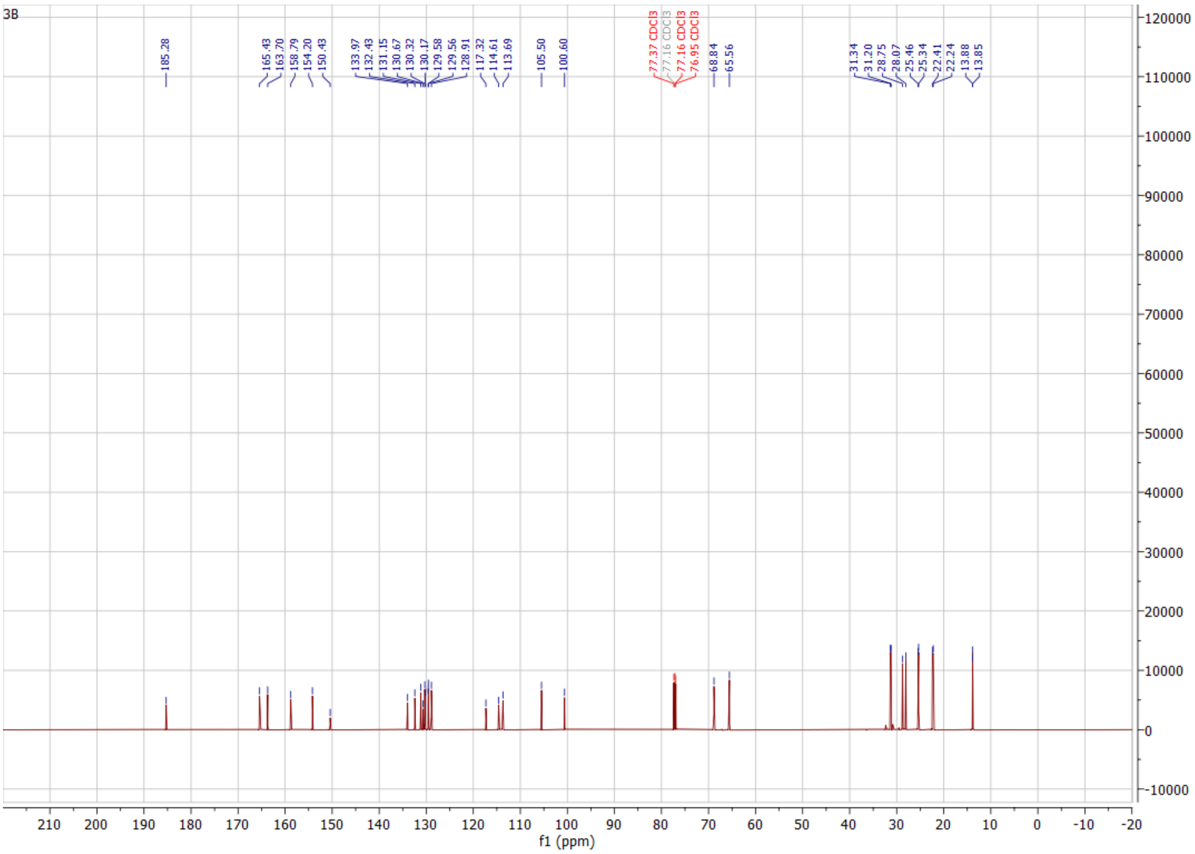

Figure S22. ^{13}C-NMR (151 MHz, chloroform-*d*) spectrum of product **3B**.

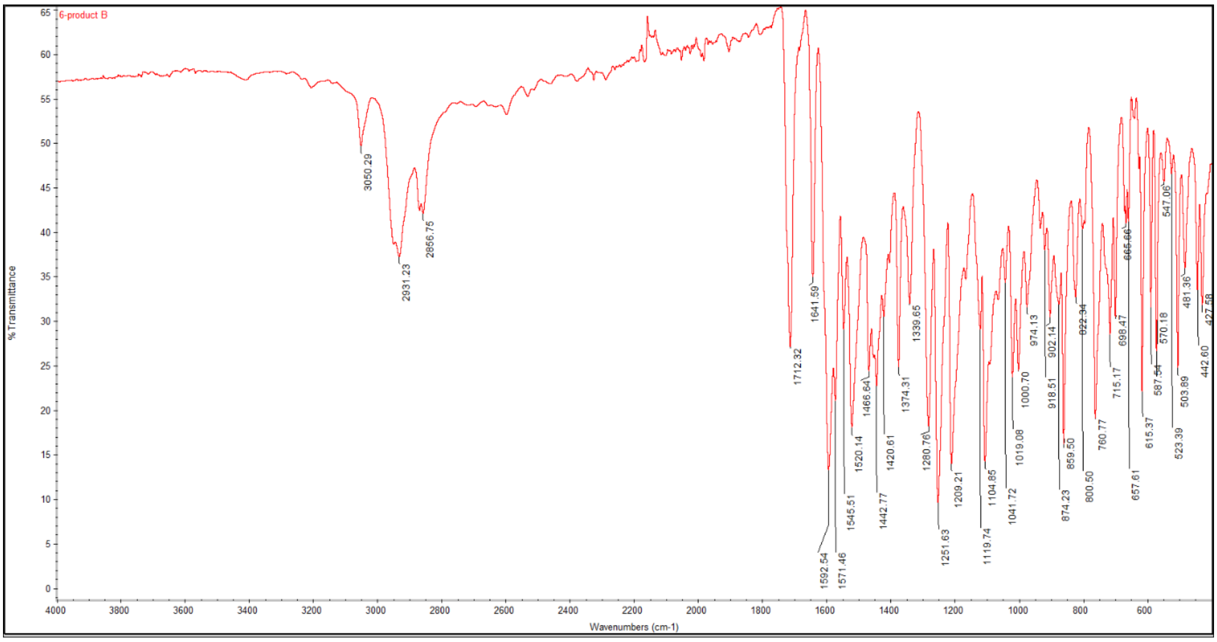

Figure S23. FTIR-ATR spectrum of product **3B**.

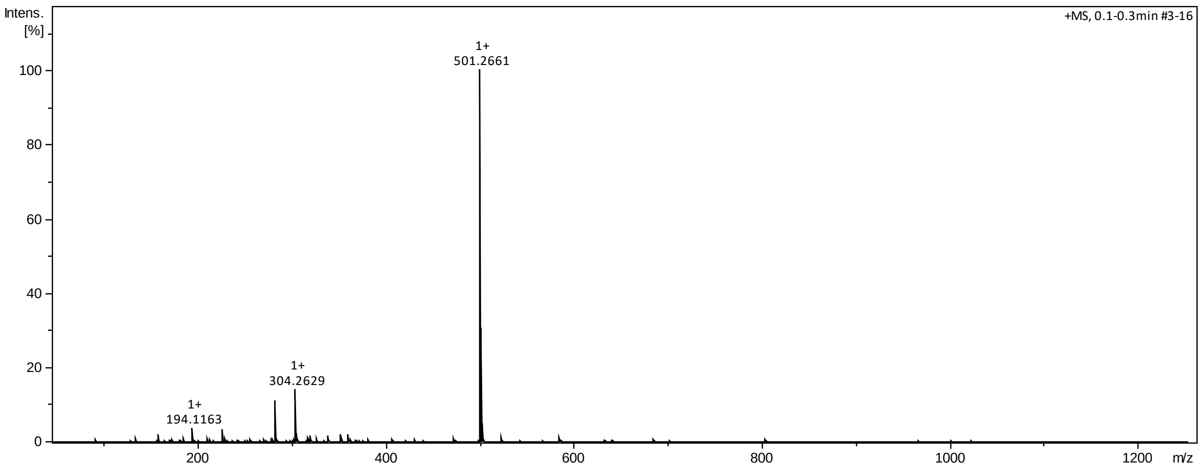

Figure S24. HR ESI-MS spectrum of product **3B**.

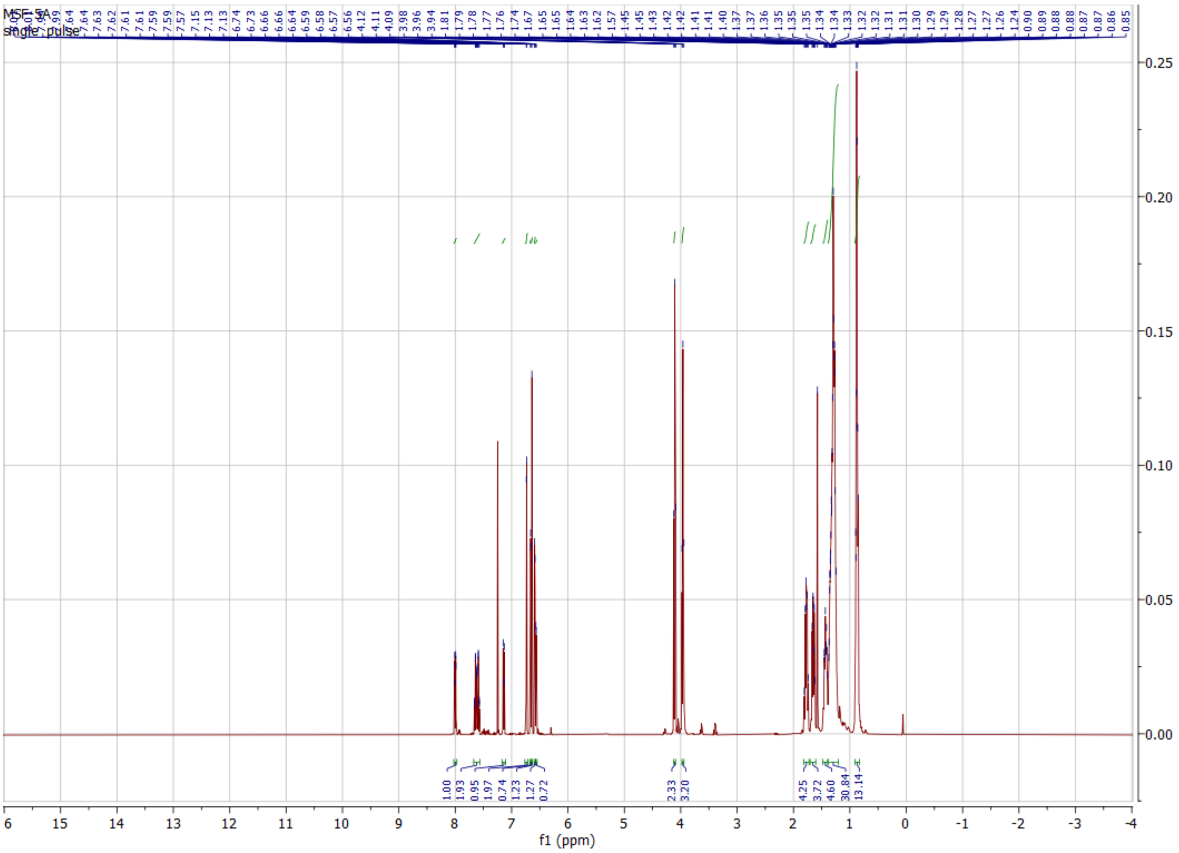

Figure S25. ^{1}H-NMR (600 MHz, chloroform-*d*) spectrum of product **4A**.

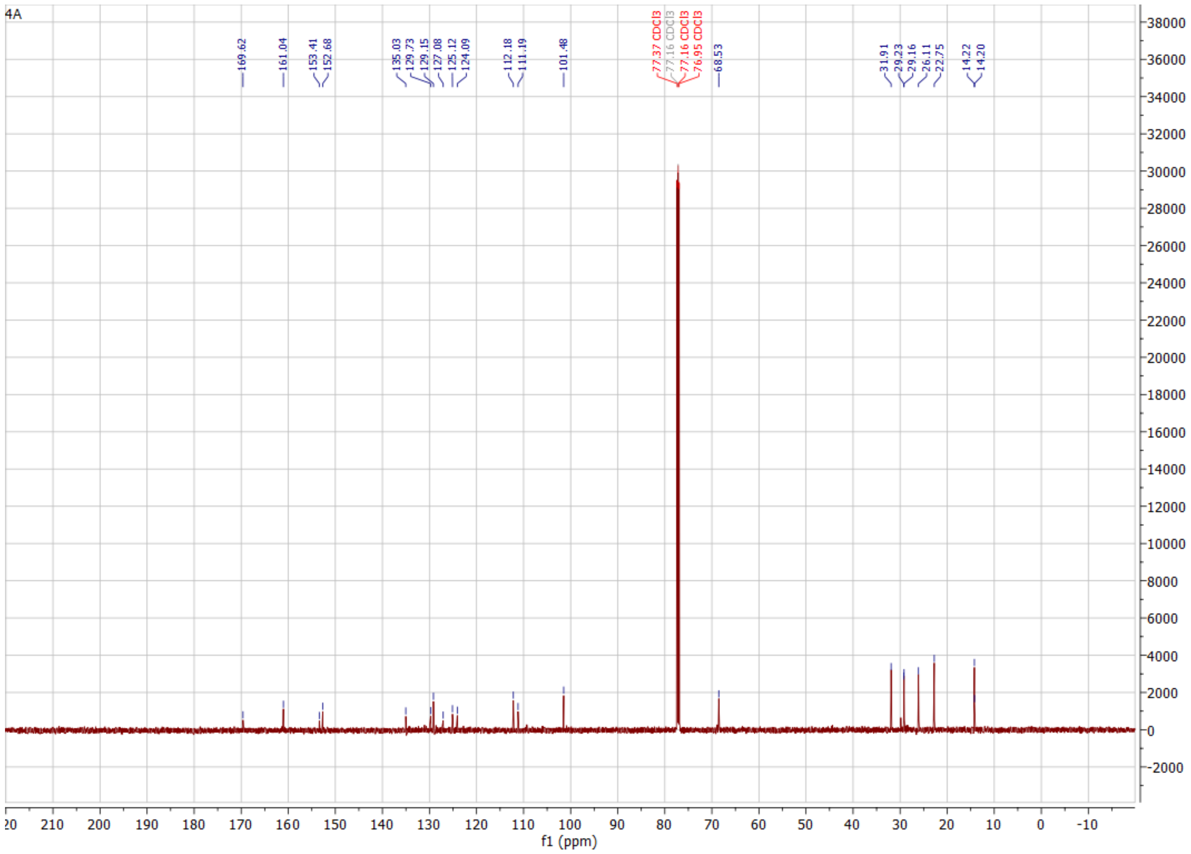

Figure S26. ^{13}C-NMR (151 MHz, chloroform-*d*) spectrum of product **4A**.

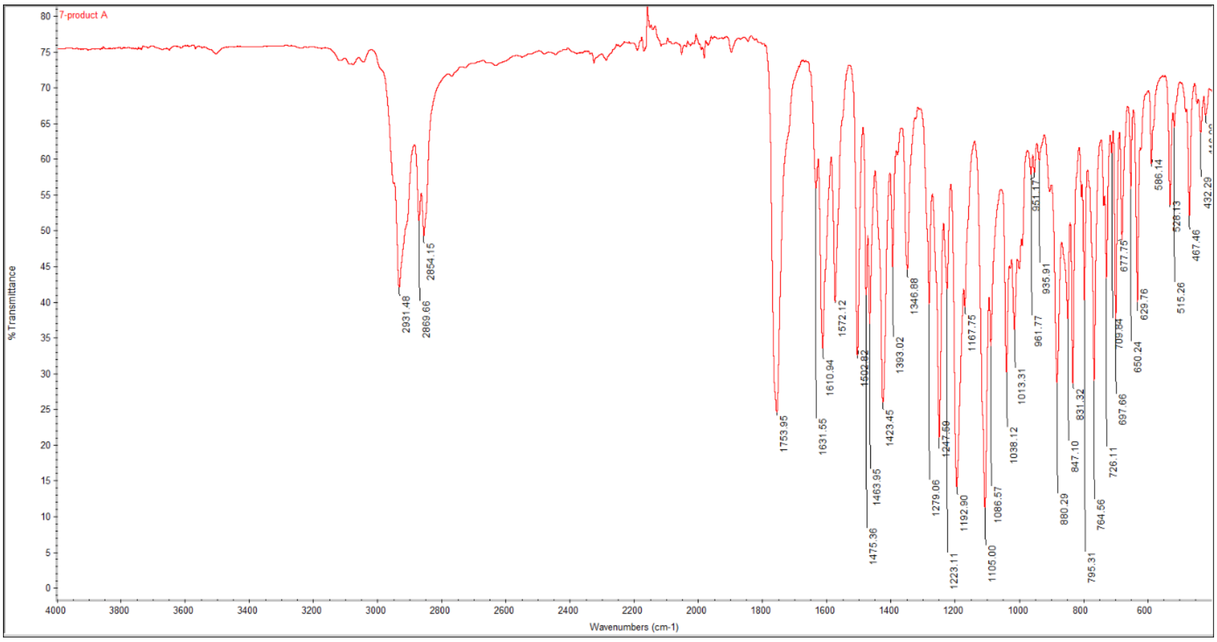

Figure S27. FTIR-ATR spectrum of product **4A**.

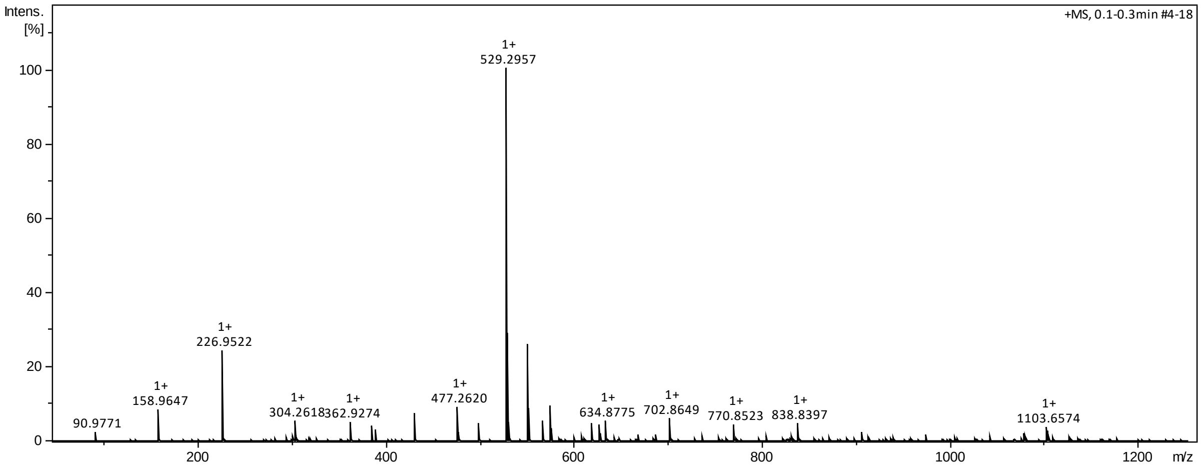

Figure S28. HR ESI-MS spectrum of product **4A**.

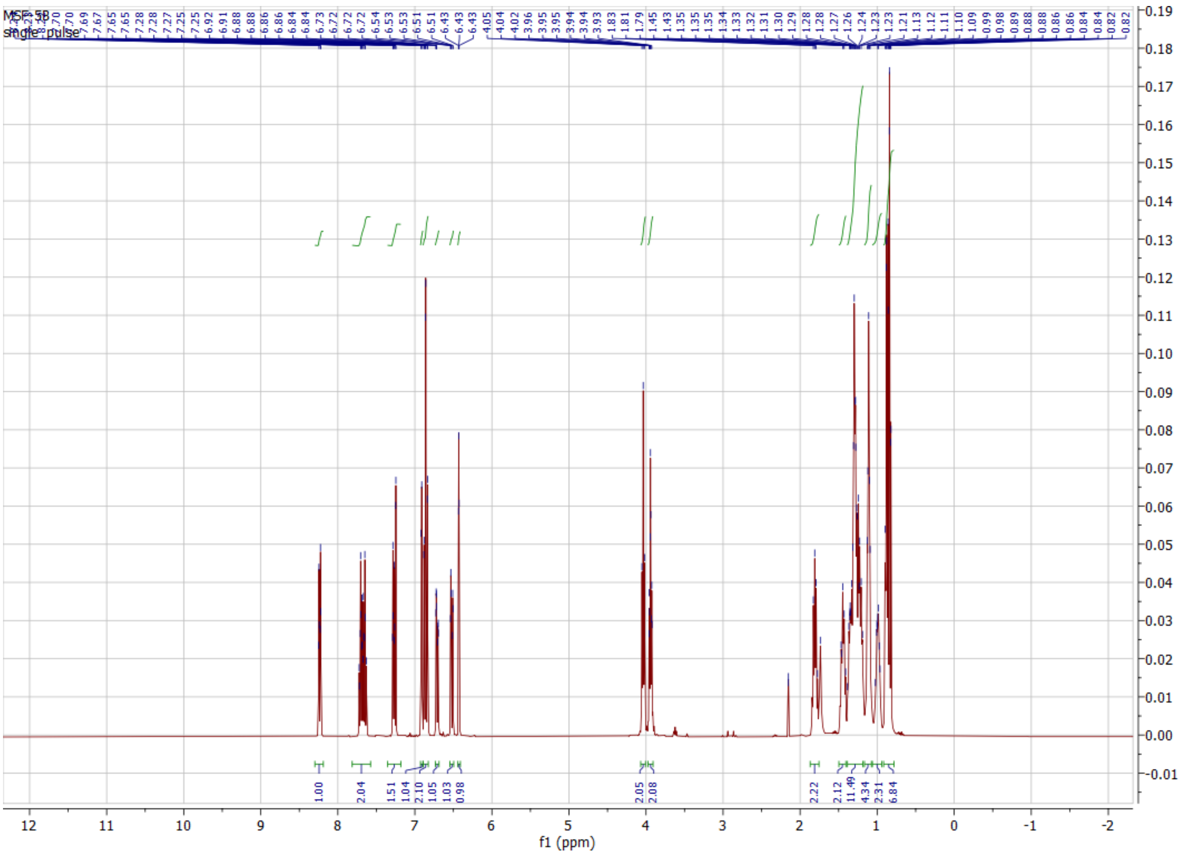

Figure S29. ^{1}H-NMR (600 MHz, chloroform-*d*) spectrum of product **4B**.

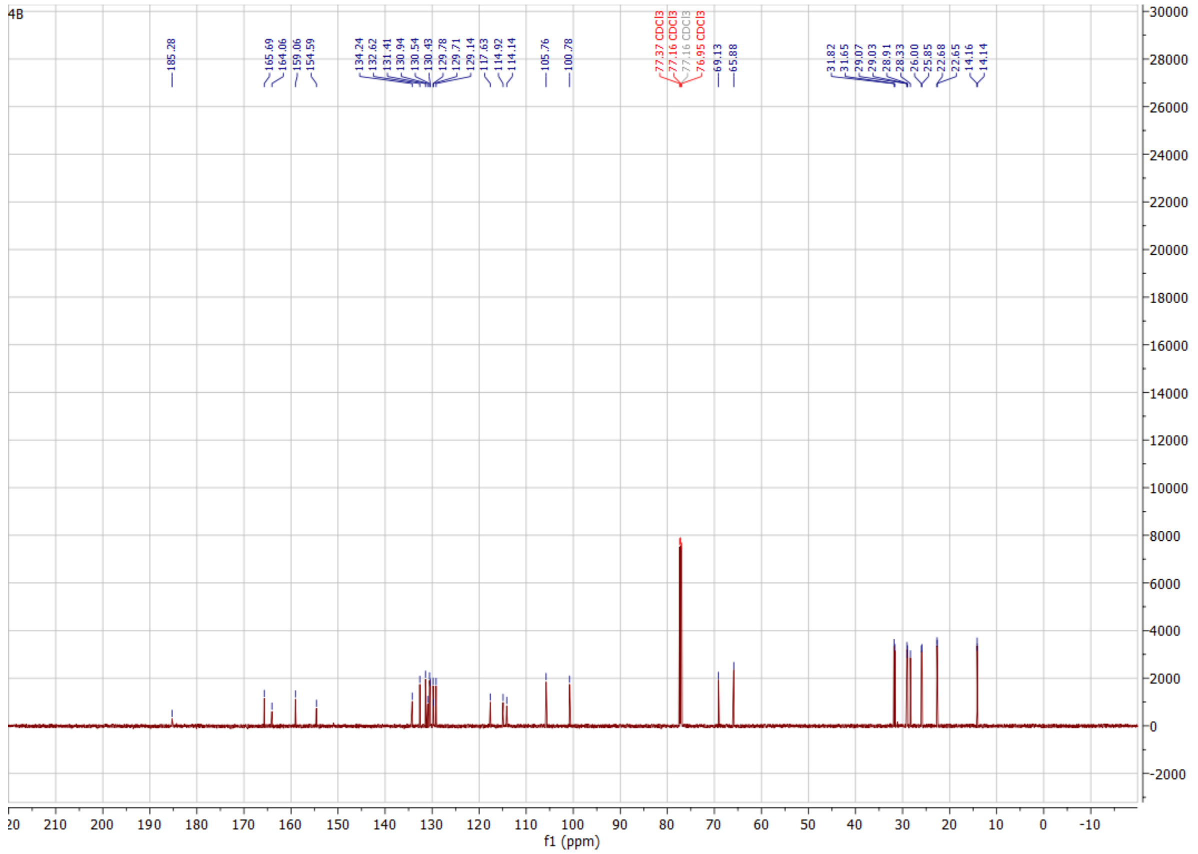

Figure S30. ^{13}C-NMR (151 MHz, chloroform-*d*) spectrum of product **4B**.

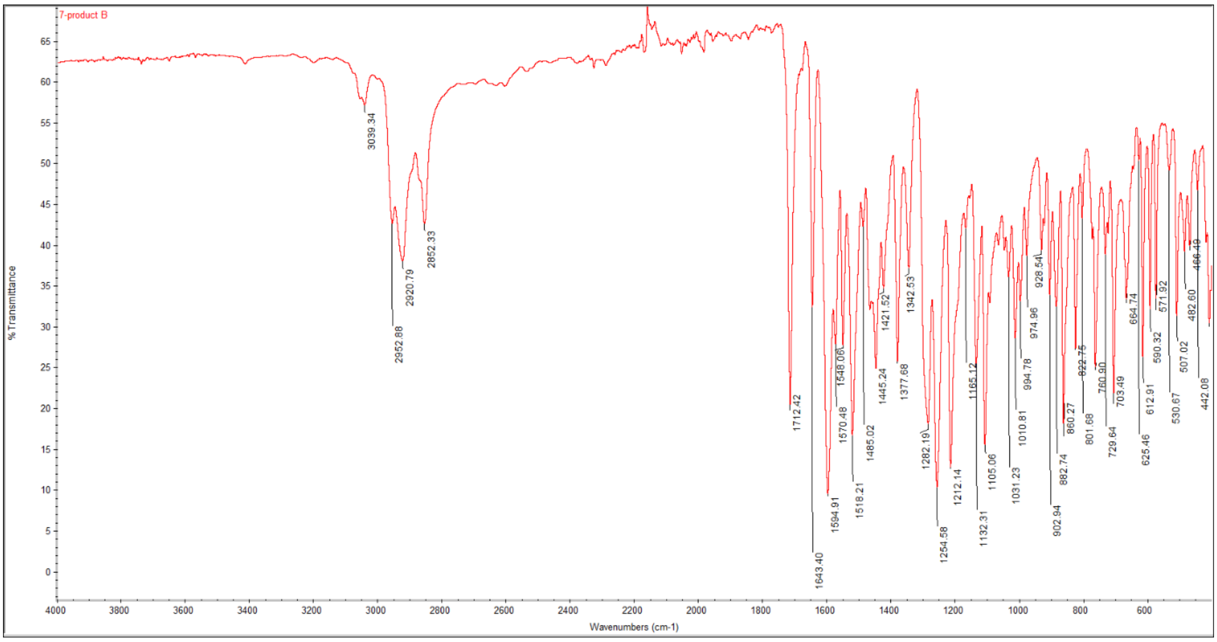

Figure S31. FTIR-ATR spectrum of product **4B**.

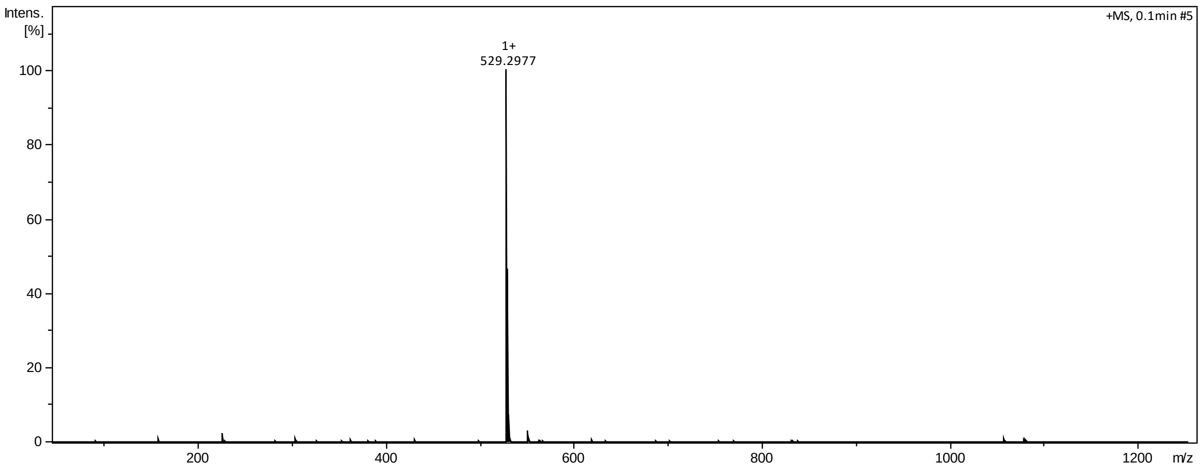

Figure S32. HR ESI-MS spectrum of product **4B**.

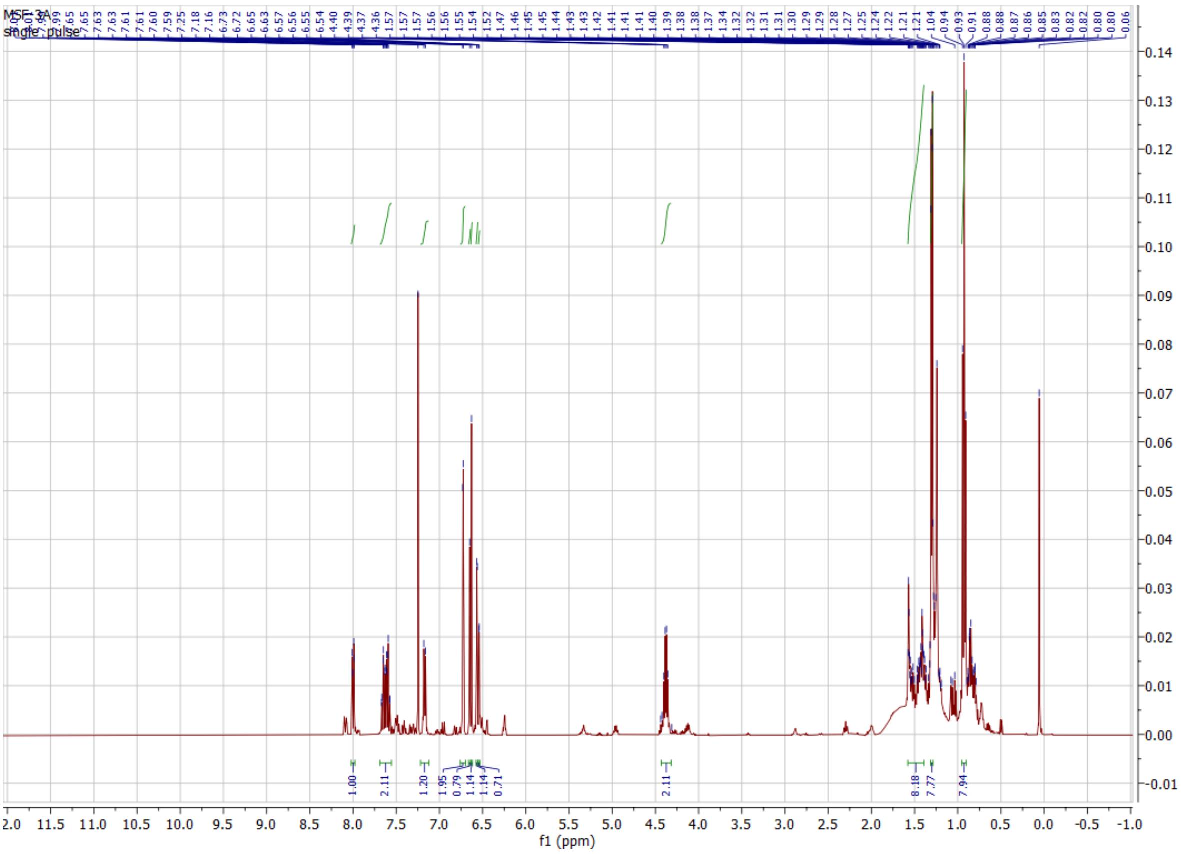

Figure S33. ^{1}H-NMR (600 MHz, chloroform-*d*) spectrum of product **5A**.

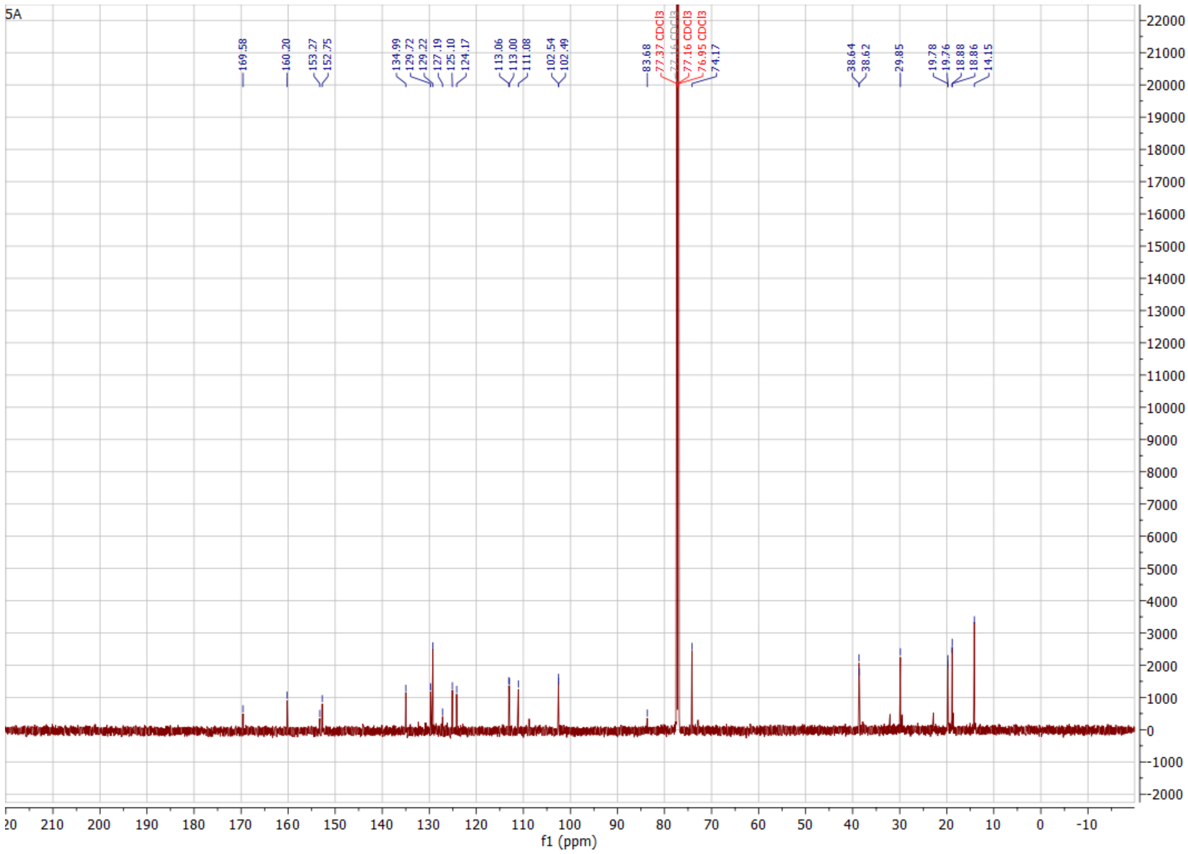

Figure S34. ^{13}C-NMR (151 MHz, chloroform-*d*) spectrum of product **5A**.

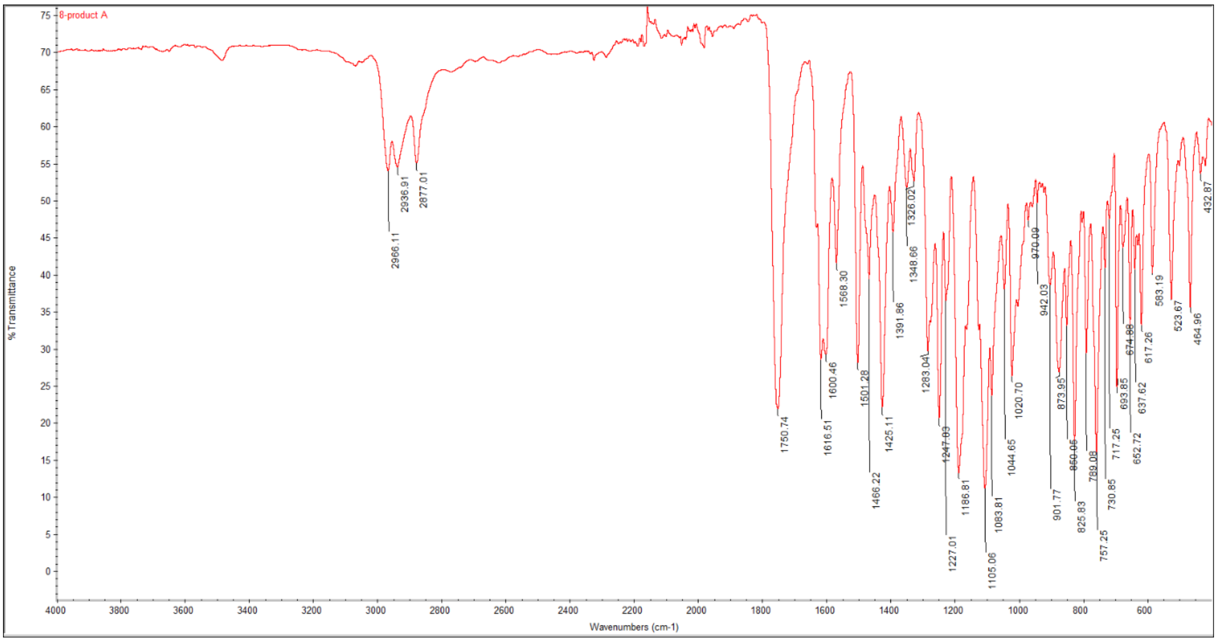

Figure S35. FTIR-ATR spectrum of product **5A**.

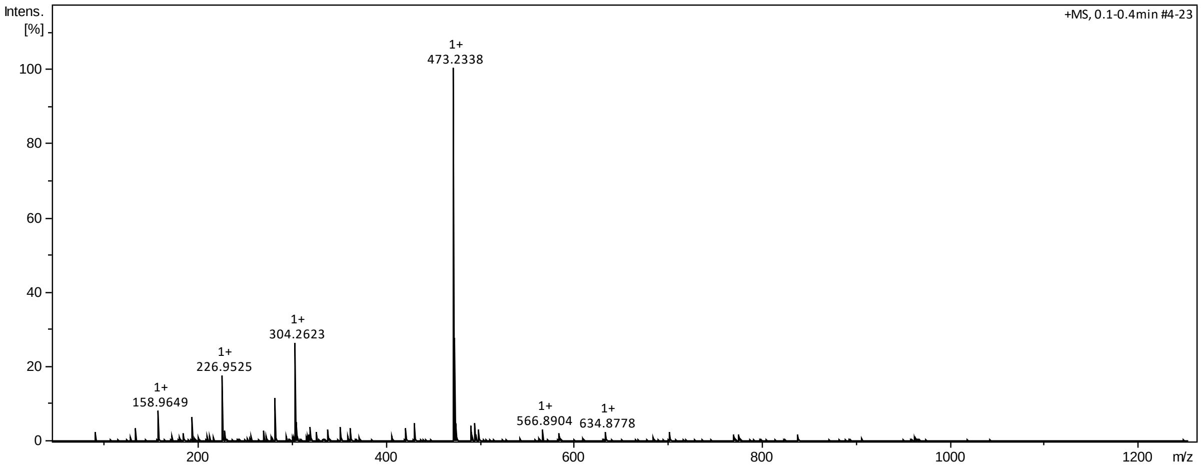

Figure S36. HR ESI-MS spectrum of product **5A**.

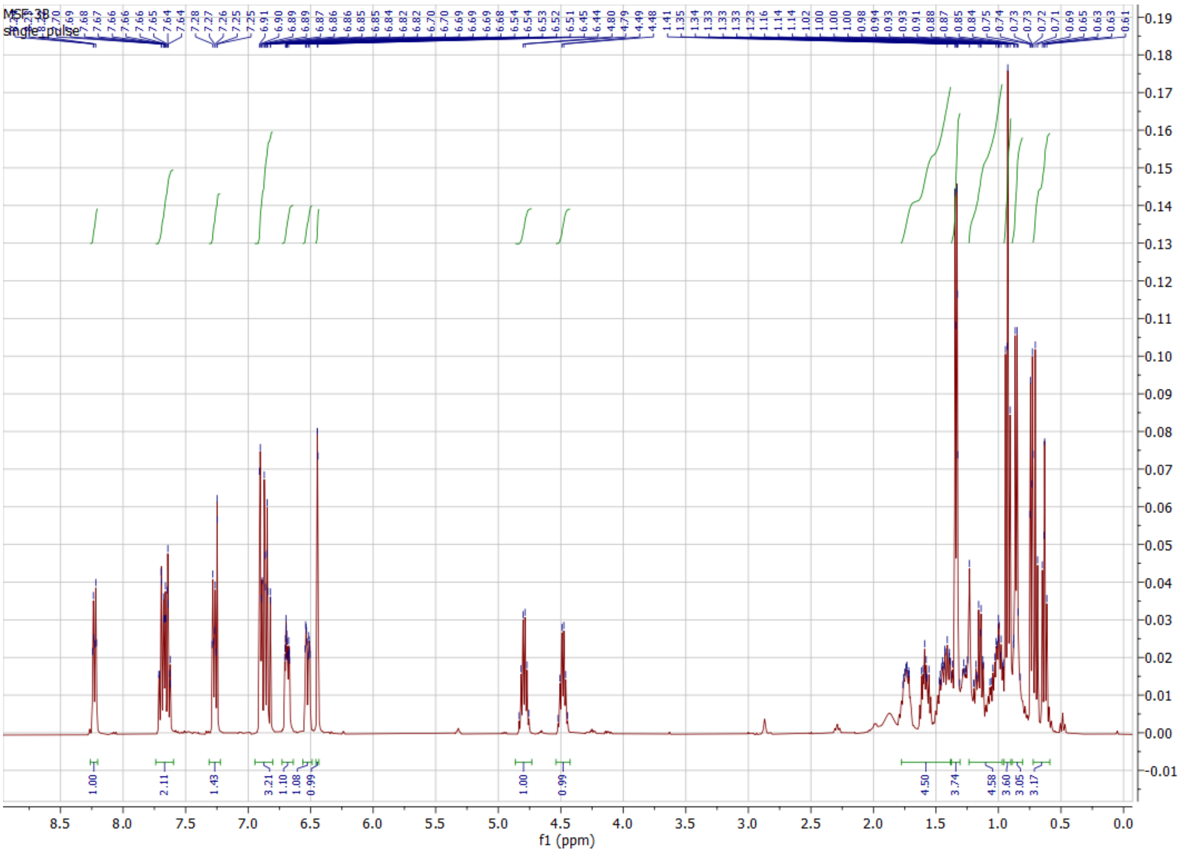

Figure S37. ^{1}H-NMR (600 MHz, chloroform-*d*) spectrum of product **5B**.

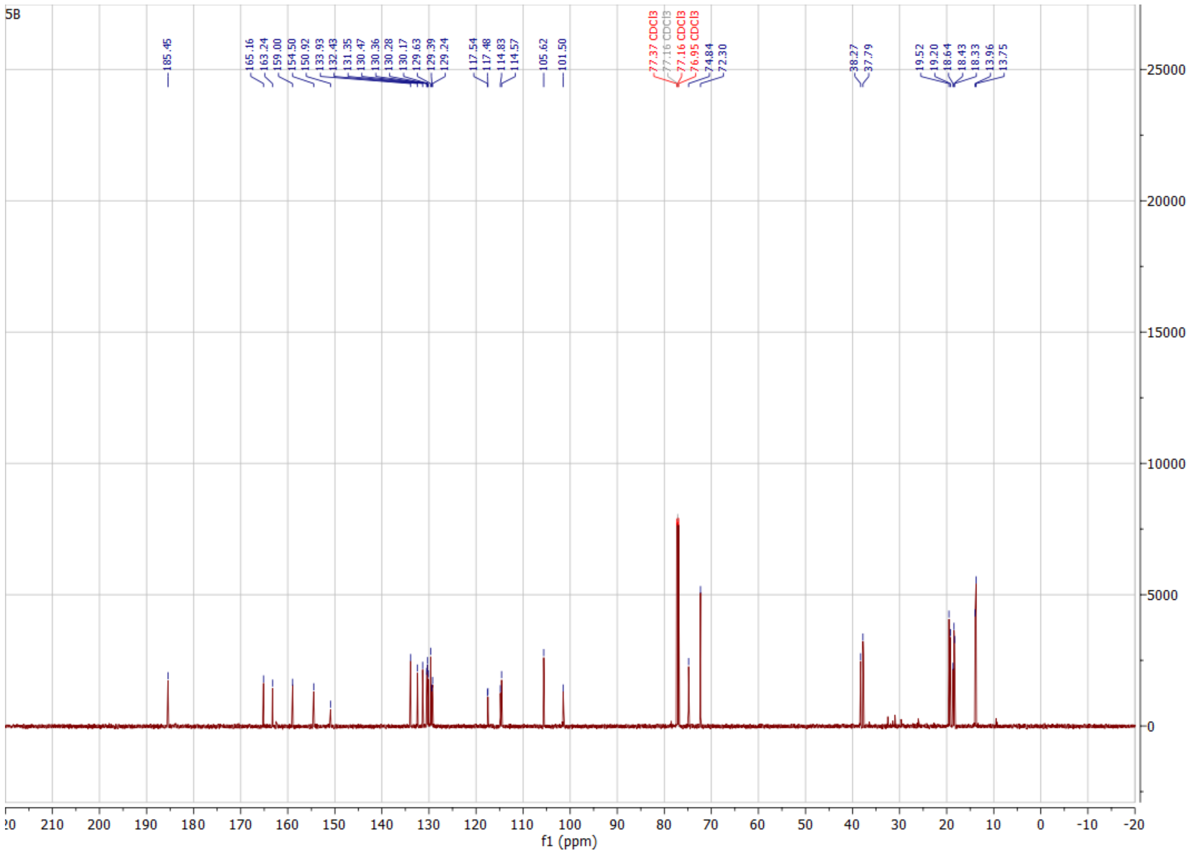

Figure S38. ^{13}C-NMR (151 MHz, chloroform-*d*) spectrum of product **5B**.

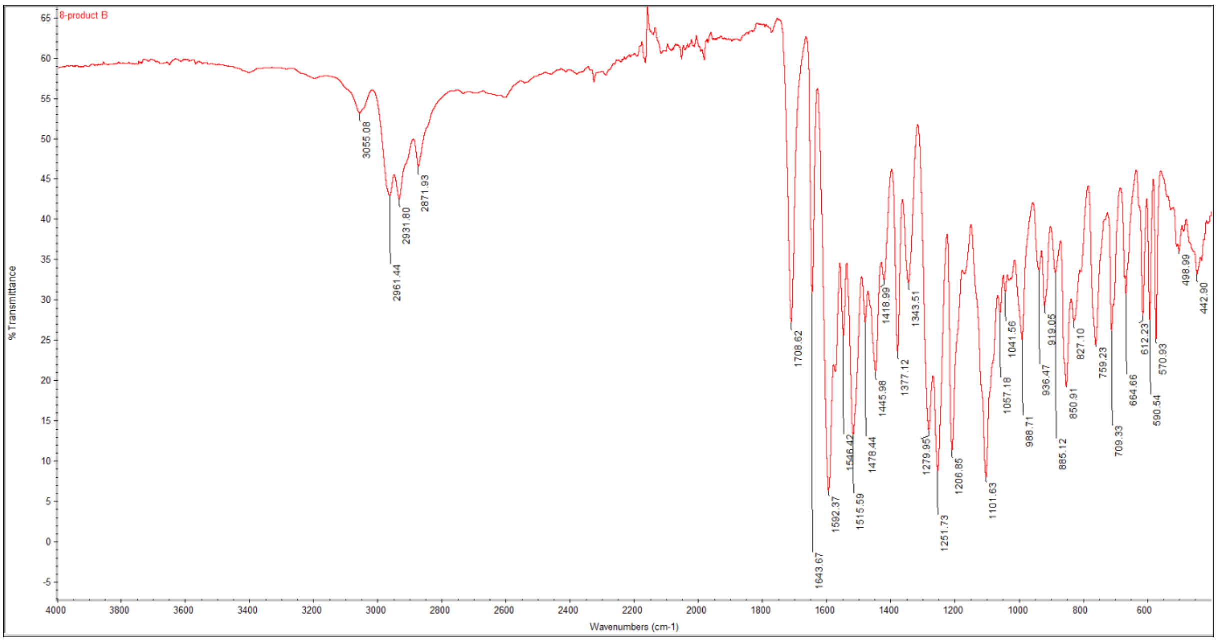

Figure S39. FTIR-ATR spectrum of product **5B**.

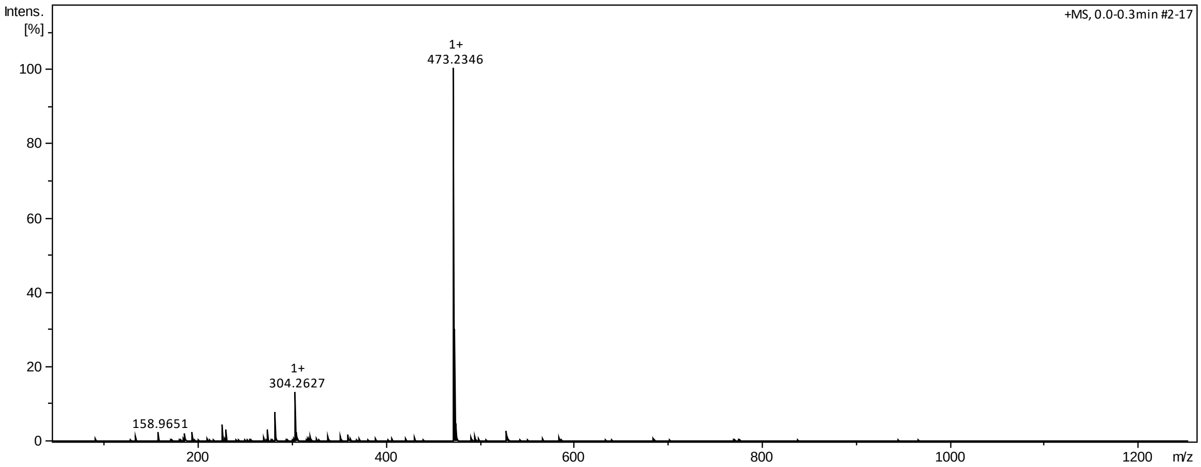

Figure S40. HR ESI-MS spectrum of product **5B**.

S2. Spectroscopic properties

Table S1. Summary of spectroscopic properties measured for all compounds.

Compound	λ_{abs} (nm)	λ_{em} (nm)	T (ns)	QY (%)
1A	445	464	4.5	20.94
2A	445	465	5.9	12.12
3A	445	462	5.6	26.83
4A	445	464	5.3	32.3
5A	442	465	5.5	29.55
1B	452 / 475	517	3.3	28.90
2B	453 / 475	515	3.2	32.14
3B	455	518	3.3	24.15
4B	455	520 / 556	3.1	36.56
5B	454 / 480	518	3.2	27.65
Parent dye – cationic form	437	475	3.5–4.4	90-100
Parent dye – neutral form	453; 474	517	2.97	29

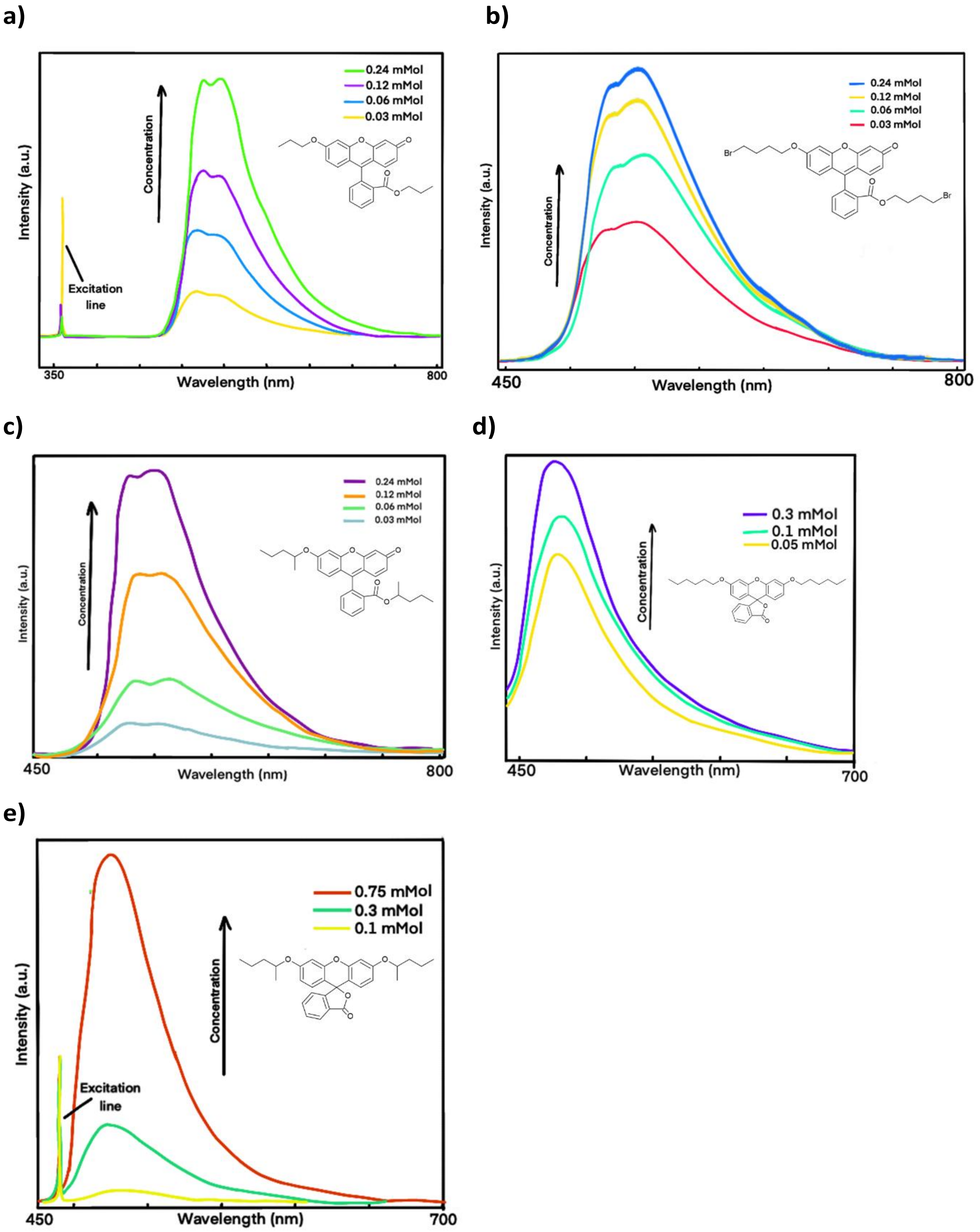

Figure S41. Emission spectra for different concentration measured for synthetized compounds (**a**) **1B**, (**b**) **2B**, (**c**) **5B**, (**d**) **3A**, (**e**) **5A**.
